# Metal-Free Photoredox Catalyzed Cyclization of *O*-(2,4-Dinitrophenyl)oximes to Phenanthridines

**DOI:** 10.3390/molecules21121690

**Published:** 2016-12-08

**Authors:** Xiubin Liu, Zhixing Qing, Pi Cheng, Xinyu Zheng, Jianguo Zeng, Hongqi Xie

**Affiliations:** 1 National and Provincial Union Engineering Research Center for the Veterinary Herbal Medicine Resources and Initiative, Hunan Agricultural University, Changsha 410128, China; Xiubin_liu@163.com (X.L.); zxqing1219@sina.com (Z.Q.); xinyuzazy@outlook.com (X.Z.); ginkgo@world-way.net (J.Z.); 2 Hunan Co-Innovation Center for Utilization of Botanicals Functional Ingredients, Hunan Agricultural University, Changsha 410128, China

**Keywords:** visible light, photoredox catalysis, eosin Y, *O*-aryl oximes, phenanthridines

## Abstract

A metal-free visible-light photoredox-catalyzed intermolecular cyclization reaction of *O*-2,4-dinitrophenyl oximes to phenanthridines was developed. In this study, the organic dye eosin Y and *i*-Pr_2_NEt were used as photocatalyst and terminal reductant, respectively. The oxime substrates were transformed into iminyl radical intermediates by single-electron reduction, which then underwent intermolecular homolytic aromatic substitution (HAS) reactions to give phenanthridine derivatives.

## 1. Introduction

Phenanthridine is important substructure found in many naturally occurring alkaloids such as sanguinarine and chelerythrine [1,2,3]. Member of this family have demonstrated pharmaceutical activities, including antitumor [4], antifungal [5], antibacterial [6,7], DNA intercalator [8,9] and enzyme inhibition [10,11]. Furthermore, phenanthridine derivatives have wide applications in materials science because of their unique optoelectronic properties [12]. For the above reasons, diverse methodologies have been established to prepare phenanthridine derivatives, such as Lautens’s palladium-catalyzed domino direct arylation/*N*-arylation of triflates [13,14] and Studer’s tandem radical addition-cyclization reaction of 2-isocyanobiphenyls with diverse radical precursors [15,16,17,18].

**Scheme 1:**
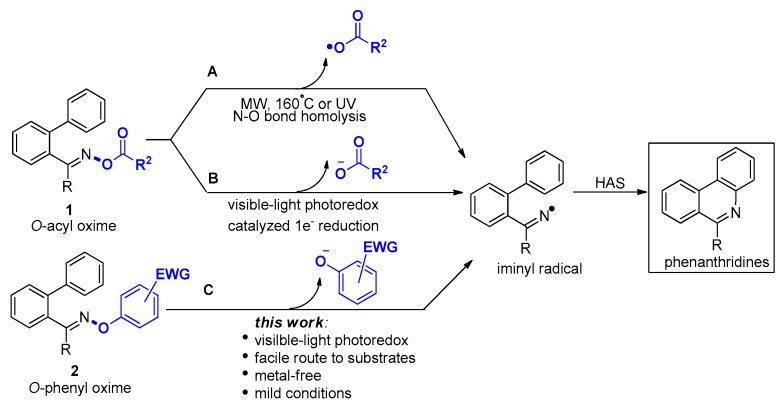
Homolysis and one e^−^ reduction of *O*-acyl and *O*-phenyl oximes to iminyl radicals.

The intramolecular homolytic aromatic substitution (HAS) reactions of iminyl radicals have shown advantages in the synthesis of phenanthridine derivatives and other *N*-containing heterocycles. One of the pathways to iminyl radicals is the N-O bond cleavage of *O*-acyl or aryl oximes under the UV or microwave irradiation at high temperature reported by Walton [19,20,21,22,23] and colleagues (Scheme 1A). Recently, s visible light photocatalytic strategy for the conversion of *N*-containing compounds though a *N*-radicals and radical ion intermediates pathway was proved to be a mild and general tool in radical reactions [24]. By taking advantage of the single-electron redox potential of a photoexcited catalyst Ir(ppy)_3_, Yu and co-workers [25,26] found that the acyl oximes could be converted to iminyl radical intermediates which were able to undergo intramolecular homolytic aromatic substitution to give phenanthridines (Scheme 1B). More recently, Leonori and co-workers developed a photoredox cyclization of iminyl [27] and amidyl radicals [28] derived from electron-poor aryloximes and aryloxy-amides, and this activation mode was applied in the synthesis of dihydropyrrole and lactam derivatives.

In the context of our study on biological active phenanthridine derivatives [11,29,30,31,32,33,34], we focused our attention on the development of facile, efficient and environmental-friendly synthetic method for phenanthridines and related compounds [35,36]. Drawing inspiration from the work of Walton, Yu and Leonori, we speculated that a visible-light photoredox catalyzed single electron reduction of electron-poor *O*-phenyl oximes **2** (Scheme 1C) to iminyl radicals might be followed by the generation of phenanthridines.

## 2. Results and Discussion

Among the commonly available electron-poor *O*-aryl oximes, *O*-(2,4-dinitrophenyl) oxime has the highest *E*_1/2_^red^ potential value of −0.55 V [27], which is suitable for SET with the excited state of commonly used photocatalysts such as Ru(bpy)_3_Cl_2_.6H_2_O (*E*_1/2_^*II/I^ = +0.77 V vs SCE), Ir(ppy)_3_ (*E*_1/2_^*III/IV^ = −1.73 V vs. SCE) and the organic dye eosin Y (*E*_1/2_^*EY/+EY^ = −1.11 V vs. SCE) [37]. In this study, as shown in Table 1, *O*-(2,4-dinitrophenyl) oxime (**3a**, Table 1) was used as model substrate. Ru(bpy)_3_Cl_2_·6H_2_O was firstly selected as photocatalyst and acetonitrile was used as solvent. After 24 h of reaction under visible light irradiation, only traces of phenanthridine (**4a**) could be detected along with the recovered starting compound **3a**. The addition of the terminal reductant *i*-Pr_2_NEt was necessary to quench the visible light excited Ru^II^* and give Ru^I^ species, a stronger electron donor (*E*_1/2_^II/I^ = −1.33 V vs. SCE) [37] that could accelerate the single electron transfer (SET) process between the substrate and photocatalyst. As it can be seen in entry 2, target compound **4a** was isolated in 32% yield when *i*-Pr_2_NEt was used. Next, DMSO (entry 3) and DMF (entry 4) were screened as reaction solvents, respectively, which demonstrated that DMF was suitable for this type of radical cyclization reaction. Further photocatalyst screening showed that the replacement of Ru(bpy)_3_Cl_2_.6H_2_O with Ir(ppy)_3_ (*E*_1/2_^*III/IV^ = −1.73 V vs. SCE) [37] could afford phenanthridine **4a** in 28% yield with part of starting material **3a** being recovered (entry 5). As we anticipated, the combined use of *i*-Pr_2_NEt and Ir(ppy)_3_ significantly increased the yield of **4a** to 51% (entry 6). It should be noted that 2-phenyl benzonitrile was detected as the major byproduct in entries 1–6. When the organic dye eosin Y (*E*_1/2_^*EY/+EY^ = −1.11 V vs. SCE) [27] was used instead of Ir(ppy)_3_, an obviously increased yield of compound **4a** was observed (75%, entry 7). The addition of *i*-Pr_2_NEt could give compound **4a** in 74% yields after 12 h of reaction (entry 8). No conversion of substrate was observed when the reaction was carried out in darkness (entry 9). Interestingly, compound **4a** was obtained in 8% yield when the reaction was carried out under visible light irradiation in the absence of photocatalyst (entry 10). According to Leonori’s studies [27], we suggested that a simple tertiary amine *i-*Pr_2_NEt would be able to reversibly interact with the 2,4-dinitrobenzene motif of **3a** to give an electron donor–acceptor complex. Visible light irradiation could initiate a SET process of this complex to give the radical ion pair which would successively undergo fragmentation to give iminyl radical. Without visible light excitation, *i*-Pr_2_NEt could not initiate the cyclization reaction (entry 11). Finally, a trace of target compound **4a** could be detected by simply heating a solvent of **3a** in DMF at 100 °C for 6 h (entry 12). The generation of trace cyclization product in entry 12 was possibly ascribed to the intermolecular nucleophilic substitution because N-O bond in **3a** was weak and the 2,4-dinitrophenoxy motif was a suitable leaving group in the substitution reaction.

Having developed a photoredox transition-metal-free radical cyclization as shown in Table 1, entry 8, we decided to explore the scope of substituent groups on the aryl ring of *O*-(2,4-dinitrophenyl)oximes **3**. As shown in Table 2, when R^1^ were electron-donating groups such as methoxyl, methyl, 2,4-dimethyl and chloro atoms, the target compounds **4b**–**4e** were isolated in moderate yields (46%–56%), which were lower than that of **4a**. Interestingly, when group R^1^ was replaced by a trifluoromethyl group, an obviously increased yield of compound **4f** was observed. It was suggested that electron-poor phenyl ring A of substrate **3** was much more suitable for the present HAS reaction. When the A ring of substrate **3** was 3-methyl)-substituted (compound **3g**) the HAS reaction provided **4ga** and **4gb** in a ratio of 2:1 with total yield of 47%. Similar experimental results could be observed when ring A was 3,4-dimethoxyl-substituted (**3h**), and target compounds **4ha** and **4hb** were isolated in 58% total yield with a ratio of 2:1. We next turned to explore the scope of substituent group R^2^ on ring B of substrate **3**. When R^2^ were 4′,5′-dimethoxy groups, target compounds **4i**–**4l** were isolated in 40%–51% yield. Further exploration showed that changing R^2^ to 4-F (**3m**–**3p**) or 4-Me (**3q**–**3r**) had no apparent effects on the yield of the phenanthridine derivatives, and target compounds **4m**–**4r** were isolated in 40%–57% yield.

**Scheme 2:**
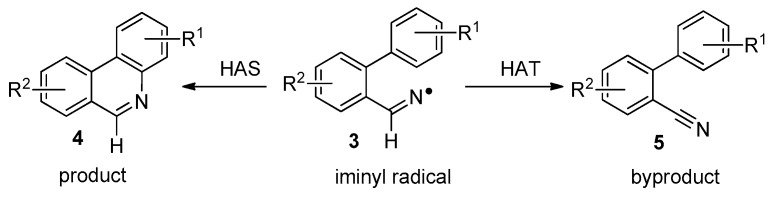
Competitive transformation of iminyl radicals through HAS and HAT process.

It was worth noting that 2-phenylbenzonitrile derivatives **5** were detected as byproducts as shown in Scheme 2. We speculate that these nitriles were produced by a competing hydrogen atom transfer (HAT) process. In order to avoid the HAT process, *O*-2,4-dinitrophenyl acetophenone oximes **6** were evaluated as substrates in the radical cyclization reaction to give corresponding 6-methyl phenanthridines **7** (Table 3).

As shown in Table 3, the metal-free photoredox-catalyzed cyclization of *O*-2,4-dinitrophenyl acetophenone oximes **6** provided 6-methylphenanthridines **7** in excellent yield (85%–92%). Yields of target compounds **7a**–**7f** were not obviously affected by the R^1^ group.

**Scheme 3:**
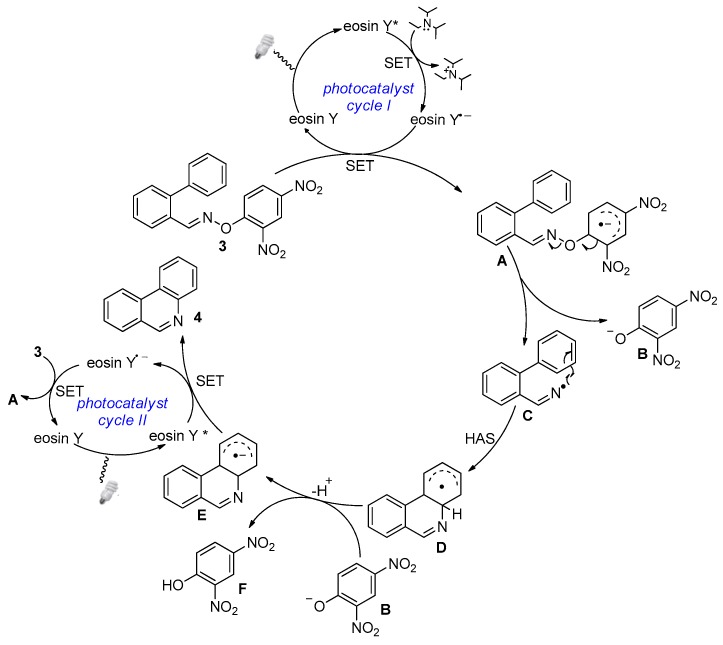
Possible mechanism of visible-light promoted cyclization of *O*-phenyl oximes.

Based on the above experimental results in this study and previous work reported by Leonori [27], a reaction mechanism could be proposed, as shown in Scheme 3. In photocatalyst cycle **I**, visible light excited eosin Y* was reduced by *i*-Pr_2_NEt to eosin Y^•−^, which was a more powerful reductant that could reduce substrate **3** to radical anion **A**. The fragmentation of radical anion **A** led to phenoxyl anion **B** and iminyl radical intermediate **C** [27]. Cyclization of radical **C** through HAS process gave radical **D** which was further deprotonated by phenoxyl anion **B** to radical anion **E**. At this stage, radical anion **E** was involved in photocatalyst cycle **II** and was oxidized by excited eosin Y* to target compound **4** along with the generation of Eosin Y**^•^**^−^ which was able to reduce substrate **3** and led to the generation of ground state eosin Y to complete photocatalyst cycle **II**.

## 3. Experimental Section

### 3.1. General Information

All reactions were carried out under a nitrogen atmosphere unless otherwise stated. ^1^H-NMR (400 MHz) and ^13^C-NMR (100 MHz) spectra were obtained at 25 °C with CDCl_3_ as solvent and TMS as internal standard on a Bruker AVANCE III 400 M NMR instrument (Bruker, Swiss). HRMS data were obtained in the ESI mode on a 6530 Q-TOF/MS system (Agilent, Singapore). For flash chromatography silica gel (200–300 mesh) was employed (Qingdao Haiyang Chemical Co., Ltd., Qingdao, China). The ^1^H-NMR and ^13^C-NMR spectrum of compounds **4** and **7** are available at the Supplementary Materials.

### 3.2. Representative Experimental Procedure for Visible Light Promoted Synthesis of Phenanthridines ***4*** and ***7***

A solution of *O*-(2,4-dinitrophenyl) oximes **3** or **6** (0.5 mmol), 1.5 eq of *i*-Pr_2_NEt, 2 mol % eosin Y in DMF (5 mL) was firstly bubbled with nitrogen for 10 min and then irradiated with a 25 W household compact fluorescent lamp. After 16 h of reaction, the resulting mixture was poured into water (50 mL) and then extracted with EtOAc (20 mL × 3). The combined organic solution was then washed with water (20 mL × 3). The organic layers were washed with brine and dried over MgSO_4_. The solvent were removed via vacuo and the residue was purified by flash column chromatography (SiO_2_) with petroleum ether/EtOAc (8:1) to give target compounds **4** or **7**.

### 3.3. Physical, Analytical and Spectral Data

*Phenanthridine* (**4a**): White amorphous powder, ^1^H-NMR: δ ppm 9.28 (s, 1H), 8.58 (dd, *J* = 10.6, 8.4 Hz, 2H), 8.20 (d, *J* = 8.0 Hz, 1H), 8.03 (d, *J* = 8.0 Hz, 1H), 7.90–7.80 (m, 1H), 7.78–7.72 (m, 1H), 7.68 (td, *J* = 8.1, 0.9 Hz, 2H).^13^C-NMR: δ ppm 153.7, 144.6, 132.7, 131.1, 130.3, 128.9, 128.8, 127.6, 127.2, 126.5, 124.2, 122.3, 122.0. HRMS (ESI^+^): calcd 180.0808 for C_13_H_10_N^+^ [M + H]^+^; found, 180.0810.

*3-Methoxyphenanthridine* (**4b**): Pale yellow amorphous powder, ^1^H-NMR: δ ppm 9.24 (s, 1H), 8.49 (t, *J* = 8.4 Hz, 1H), 8.45 (d, *J* = 8.8 Hz, 1H), 8.00 (d, *J* = 8.0 Hz, 1H), 7.82–7.80 (m, 1H), 7.64–7.60 (m, 2H), 7.31 (dd, *J* = 8.8, 2.4 Hz, 1H), 3.98 (s, 3H).^13^C-NMR: δ ppm 160.2, 154.1, 146.2, 132.9, 131.2, 128.9, 126.5, 125.7, 123.5, 121.5, 118.3, 118.2, 110.1, 55.7. HRMS (ESI^+^): calcd. 210.0913 for C_14_H_12_NO^+^ [M + H]^+^; found, 210.0918.

*3-Methylphenanthridine* (**4c**): Pale yellow amorphous powder, ^1^H-NMR: δ ppm 9.28 (s, 1H), 8.60 (d, *J* = 8.0 Hz, 1H), 8.49 (d, *J* = 8.4 Hz, 1H), 8.05 (d, *J* = 8.0 Hz, 1H), 8.01 (s, 1H), 7.87 (td, *J* = 1.6, 7.2 Hz, 1H), 7.70 (t, *J* = 7.2 Hz, 1H), 7.54 (dd, *J* = 1.6, 8.4 Hz, 1H), 2.63 (s, 3H). ^13^C-NMR: δ ppm 153.6, 144.6, 138.9, 132.7, 131.0, 129.6, 128.8, 128.7, 127.7, 126.2, 122.0, 121.7, 121.7 (overlapped), 21.6. HRMS (ESI^+^): calcd. 194.0964 for C_14_H_12_N^+^ [M + H]^+^; found, 194.0967.

*2,4-Dimethylphenanthridine* (**4d**): Pale yellow amorphous powder, ^1^H-NMR: δ ppm 9.27 (s, 1H), 8.60 (d, *J* = 8.4 Hz, 1H), 8.23 (s, 1H), 8.04 (d, *J* = 8.0 Hz, 1H), 7.83 (t, *J* = 8.0 Hz, 1H), 7.69 (t, *J* = 8.0 Hz, 1H), 7.46 (s, 1H), 2.88 (s, 3H), 2.60 (s, 3H). ^13^C-NMR: δ ppm 151.3, 141.6, 137.3, 136.4, 132.6, 131.3, 130.5, 128.6, 127.1, 126.2, 123.8, 122.0, 119.7, 21.9, 18.6. HRMS (ESI^+^): calcd. 208.1121 for C_15_H_14_N^+^ [M + H]^+^; found, 208.1117.

*3-Chorophenanthridine* (**4e**): White amorphous powder, ^1^H-NMR: δ ppm 9.30 (s, 1H), 8.56 (d, *J* = 8.4 Hz, 1H), 8.50 (d, *J* = 8.8 Hz, 1H), 8.19 (d, *J* = 2.0 Hz, 1H), 8.07 (d, *J* = 8.0 Hz, 1H), 7.90 (td, *J* = 1.2, 7.2 Hz, 1H), 7.75 (t, *J* = 8.0 Hz, 1H), 7.65 (dd, *J* = 2.4, 8.8 Hz, 1H), 7.49 (d, *J* = 7.6 Hz, 1H). ^13^C-NMR: δ ppm 154.7, 145.1, 134.3, 131.4, 130.1, 129.4, 129.0, 127.8, 127.7, 126.3, 123.6, 122.6, 121.8. HRMS (ESI^+^): calcd 214.0418 for C_13_H_9_ClN^+^ [M + H]^+^; found, 214.0414.

*3-(Trifluoromethyl)phenanthridine* (**4f**): Pale yellow amorphous powder, ^1^H-NMR: δ ppm 9.38 (s, 1H), 8.70 (d, *J* = 8.4 Hz, 1H), 8.66 (d, *J* = 8.4 Hz, 1H), 8.50 (s, 1H), 8.22 (d, *J* = 7.6 Hz, 1H), 7.96 (td, *J* = 1.6, 7.2 Hz, 1H), 7.90 (dd, *J* = 1.6, 8.8 Hz, 1H), 7.83 (td, *J* = 1.2, 8.0 Hz, 1H). ^13^C-NMR: δ ppm 154.9, 143.8, 131.8, 131.6, 130.5(q, ^2^*J*_F-C_ = 32.5 Hz), 129.0, 128.7, 127.6 (q, ^3^*J*_F-C_ = 4.2 Hz), 126.9, 126.4, 123.3 (q, ^1^*J*_F-C_ = 276.0 Hz), 123.0 (q, ^3^*J*_F-C_ = 3.2 Hz), 122.9, 122.2. HRMS (ESI^+^): calcd 248.06828 for C_14_H_9_F_3_N^+^ [M + H]^+^; found, 248.0682.

*2-Methylphenanthridine* (**4ga**): Pale yellow amorphous powder, ^1^H-NMR: δ ppm 9.26 (s, 3H), 8.63 (d, *J* = 8.4 Hz, 1H), 8.39 (s, 1H), 8.11 (d, *J* = 8.0 Hz, 1H), 8.06 (d, *J* = 8.0 Hz, 1H), 7.88 (td, *J* = 1.2, 7.2 Hz, 1H), 7.73 (t, *J* = 7.2 Hz, 1H), 7.60 (dd, *J* = 1.6, 8.4 Hz, 1H), 2.67 (s, 3H). ^13^C-NMR: δ ppm 152.6, 142.8, 142.5, 137.0, 132.4, 130.8, 130.4, 130.4 (overlapped), 129.8, 128.7, 121.8, 121.8 (overlapped), 22.0. HRMS (ESI^+^): calcd 194.0964 for C_14_H_12_N^+^ [M + H]^+^; found, 194.0963.

*4-Methylphenanthridine* (**4gb**): Pale yellow amorphous powder, ^1^H-NMR: δ ppm 9.34 (s, 1H), 8.63 (d, *J* = 8.0 Hz, 1H), 8.47 (d, *J* = 7.6 Hz, 1H), 8.08 (d, *J* = 7.6 Hz, 1H), 7.87 (t, *J* = 7.2 Hz, 1H), 7.72 (t, *J* = 7.6 Hz, 1H), 7.63–7.58 (m, 2H), 2.92 (s, 3H). ^13^C-NMR: δ ppm 152.2, 143.2, 137.8, 132.9, 130.8, 129.5, 128.7, 127.3, 126.7, 126.2, 124.0, 122.1, 120.1, 18.7. HRMS (ESI^+^): calcd 194.0964 for C_14_H_12_N^+^ [M + H]^+^; found, 194.0958.

*2,3-Dimethoxylphenanthridine* (**4ha**): White amorphous powder, ^1^H-NMR: δ ppm 9.17 (s, 1H), 8.46 (d, *J* = 8.0 Hz, 1H), 8.02 (d, *J* = 7.6 Hz, 1H), 7.84 (s, 1H), 7.82 (t, *J* = 7.6 Hz, 1H), 7.64 (t, *J* = 7.6 Hz, 1H), 7.60 (s, 1H), 4.12 (brs, 3H), 4.08 (brs, 3H). ^13^C-NMR: δ ppm 151.4, 150.9, 149.6, 140.6, 132.1, 130.5, 128.8, 126.4, 125.7, 121.3, 118.3, 110.0, 101.8, 56.1, 56.1 (overlapped). HRMS (ESI^+^): calcd 240.1019 for C_15_H_14_NO_2_^+^ [M + H]^+^; found, 240.1015.

*3,4-Dimethoxylphenanthridine* (**4hb**): White amorphous powder, ^1^H-NMR: δ ppm 9.33 (s, 1H), 8.54 (d, *J* = 8.4 Hz, 1H), 8.33 (d, *J* = 8.8 Hz, 1H), 8.05 (d, *J* = 7.6 Hz, 1H), 7.85 (brt, *J* = 7.2 Hz, 1H), 7.68 (brt, *J* = 7.6 Hz, 1H), 7.44 (d, *J* = 9.2 Hz, 1H), 4.18 (s, 3H), 4.08 (s, 3H). ^13^C-NMR: δ ppm 153.8, 152.0, 144.6, 139.6, 132.8, 131.1, 128.9, 126.8, 125.5, 121.6, 119.3, 117.7, 113.9, 62.1, 56.7. HRMS (ESI^+^): calcd 240.1019 for C_15_H_14_NO_2_^+^ [M + H]^+^; found, 240.1020.

*8,9-Dimethoxylphenanthridine* (**4i**): White amorphous powder, ^1^H-NMR: δ ppm 9.14 (s, 1H), 8.43 (dd, *J* = 1.2, 8.4 Hz, 1H), 8.16 (dd, *J* = 0.8, 8.0 Hz, 1H), 7.86 (s, 1H), 7.70 (td, *J* = 1.2, 7.2 Hz, 1H), 7.64 (td, *J* = 1.2, 7.2 Hz, 1H) 7.34 (s, 1H). 4.14 (s, 3H), 4.07 (s, 3H). ^13^C-NMR: δ ppm 152.9, 151.7, 150.0, 144.0, 130.1, 128.2, 127.8, 126.6, 123.8, 122.0, 121.7, 107.8, 101.8, 56.2, 56.1. HRMS (ESI^+^): calcd 240.1019 for C_15_H_14_NO_2_^+^ [M + H]^+^; found, 240.1016.

*3,8,9-Trimethoxyphenanthridine* (**4j**): White amorphous powder, ^1^H-NMR: δ ppm 9.10 (s, 1H), 8.31 (d, *J* = 8.8 Hz, 1H), 7.76 (s, 1H), 7.56 (d, *J* = 2.4 Hz, 1H),7.31 (s, 1H), 7.29–7.27 (m, 1H), 4.13 (s, 3H), 4.06 (s, 3H), 3.99 (s, 3H). ^13^C-NMR: δ ppm 159.4, 153.1, 152.0, 149.3, 145.5, 128.6, 122.9, 120.8, 118.0, 117.9, 109.6, 107.7, 101.3, 56.1, 56.0, 55.5. HRMS (ESI^+^): calcd 270.1125 for C_16_H_16_NO_3_^+^ [M + H]^+^; found, 270.1127.

*8,9-Dimethoxy-3-methylphenanthridine* (**4k**): White amorphous powder, ^1^H-NMR: δ ppm 9.14 (s, 1H), 8.35 (d, *J* = 8.4 Hz, 1H), 7.96 (s, 1H), 7.87 (s, 1H), 7.59 (d, *J* = 8.4 Hz, 1H), 7.36 (s, 1H), 4.16 (s, 3H), 4.09 (s, 3H), 2.61 (s, 3H). ^13^C-NMR: δ ppm 152.9, 151.7, 149.7, 144.1, 137.9, 129.6, 128.8, 128.4, 128.3, 121.6, 121.5, 107.8, 101.7, 56.2, 56.1, 21.5. HRMS (ESI^+^): calcd 254.1176 for C_16_H_16_NO_2_^+^ [M + H]^+^; found, 254.1172.

*3-Fluoro-8,9-dimethoxyphenanthridine* (**4l**): White amorphous powder, ^1^H-NMR: δ ppm 9.17 (s, 1H), 8.44 (dd, *J* = 6.0, 8.8 Hz, 1H), 7.84 (s, 1H), 7.81 (dd, *J* = 2.8, 10.0 Hz, 1H), 7.45–7.40 (m, 1H), 7.38 (s, 1H), 4.16 (s, 3H), 4.09 (s, 3H). ^13^C-NMR: δ ppm 162.1 (d, ^1^*J*_F-C_ = 245.8 Hz), 153.3, 152.9, 149.9, 145.2 (d, ^3^*J*_F-C_ = 11.7 Hz), 128.2, 123.6 (d, ^3^*J*_F-C_ = 9.4 Hz), 121.4, 120.6, 115.8 (d, ^2^*J*_F-C_ = 23.8 Hz), 114.4 (d, ^2^*J*_F-C_ = 20.4 Hz), 107.9, 101.6, 56.2, 56.1. HRMS (ESI^+^): calcd 258.0925 for C_15_H_13_FNO_2_^+^ [M + H]^+^; found, 258.0927.

*9-Fluorophenanthridine* (**4m**): Pale yellow amorphous powder, ^1^H-NMR: δ ppm 9.27 (s, 1H), 8.47 (d, *J* = 8.0 Hz, 1H), 8.24–8.21 (m, 2H), 8.09 (dd, *J* = 6.0, 8.8 Hz, 1H), 7.81 (td, *J* = 1.6, 8.4 Hz, 1H), 7.72 (td, *J* = 1.2, 8.0 Hz, 1H), 7.47 (td, *J* = 2.4, 8.4 Hz, 1H). ^13^C-NMR: δ ppm 164.2 (d, ^1^*J*_F-C_ = 251.0 Hz), 152.6, 144.5, 134.8 (d, ^3^*J*_F-C_ =9.5 Hz), 131.5 (d, ^3^*J*_F-C_ = 9.7 Hz), 130.2, 129.4, 127.2, 123.6, 123.4, 122.4, 116.8 (d, ^2^*J*_F-C_ = 24.2 Hz), 107.2 (^2^*J*_F-C_ = 22.5 Hz). HRMS (ESI^+^): calcd 198.0714 for C_13_H_9_FN^+^ [M + H]^+^; found, 198.0710.

*3-Methoxy-9-fluorophenanthridine* (**4n**): White amorphous powder, ^1^H-NMR: δ ppm 9.21 (s, 1H), 8.32 (d, *J* = 8.8 Hz, 1H), 8.09–8.01 (m, 2H), 7.61 (d, *J* = 2.4 Hz, 1H), 7.37–7.31 (m, 2H), 4.00 (s, 3H). ^13^C-NMR: δ ppm 164.4 (d, ^1^*J*_F-C_ = 251.0 Hz), 1606, 152.9, 145.9, 135.0 (^3^*J*_F-C_ = 8.9Hz), 131.7 (^3^*J*_F-C_ = 9.8Hz), 123.6, 122.5, 118.3, 117.7, 115.8 (^2^*J*_F-C_ = 24.2 Hz), 109.7, 106.5 (^2^*J*_F-C_ = 22.5 Hz), 55.6. HRMS (ESI^+^): calcd 228.0819 for C_14_H_11_FNO^+^ [M + H]^+^; found, 228.0819.

*9-Fluoro-3-methylphenanthridine* (**4o**): White amorphous powder, ^1^H-NMR: δ ppm 9.23 (s, 1H), 8.35 (d, *J* = 8.4 Hz, 1H), 8.17 (dd, *J* = 2.0, 10.4 Hz, 1H), 8.05 (dd, *J* = 8.4, 6.0 Hz, 1H), 8.00 (s, 1H), 7.54 (dd, *J* = 1.2, 8.0 Hz, 1H), 7.44 (dd, *J* = 2.4, 8.4 Hz, 1H), 2.63 (s, 3H). ^13^C-NMR: δ 164.2 (d, ^1^*J*_F-C_ = 250.3 Hz), 152.6, 144.6, 139.7, 134.9, 131.5 (d, ^3^*J*_F-C_ = 9.6 Hz), 129.7, 128.9, 123.1, 122.2, 121.3, 116.3 (d, ^2^*J*_F-C_ = 241 Hz), 106.9 (d, ^2^*J*_F-C_ = 22.1 Hz), 21.6. HRMS (ESI^+^): calcd 212.0870 for C_14_H_11_FN^+^ [M + H]^+^; found, 212.0859.

*3,9-Difluorophenanthridine* (**4p**): White amorphous powder, ^1^H-NMR: δ ppm 9.25 (s, 1H), 8.42 (dd, *J* = 5.6, 9.2 Hz, 1H), 8.12 (dd, *J* = 2.4, 10.4 Hz, 1H), 8.07 (dd, *J* = 5.6, 8.8 Hz, 1H), 7.84 (dd, *J* = 2.4, 9.2 Hz, 1H), 7.48–7.42 (m, 2H). ^13^C-NMR: δ ppm 164.4 (d, ^1^*J*_F-C_ = 251.0 Hz), 163.0 (d, ^1^*J*_F-C_ = 247.9 Hz), 153.8, 145.8 (d, ^3^*J*_F-C_ = 11.9 Hz), 134.5 (d, ^3^*J*_F-C_ = 9.5 Hz), 131.7 (d, ^3^*J*_F-C_ = 10.2 Hz), 124.3 (d, ^3^*J*_F-C_ = 9.5 Hz), 123.0, 120.3, 116.7 (d, ^2^*J*_F-C_ = 24.2 Hz), 116.3 (d, ^2^*J*_F-C_ = 23.8 Hz), 114.8 (d, ^2^*J*_F-C_ = 20.6 Hz), 107.0 (d, ^2^*J*_F-C_ = 22.4 Hz). HRMS (ESI^+^): calcd 216.0619 for C_13_H_8_F_2_N^+^ [M + H]^+^; found, 216.0617.

*9-Methylphenanthridine* (**4q**): Pale yellow amorphous powder, ^1^H-NMR): δ ppm 9.26 (s, 1H), 8.69 (d, *J* = 8.0 Hz, 1H), 8.42 (s, 1H), 8.20 (d, *J* = 8.4 Hz, 1H), 7.96 (d, *J* = 8.4 Hz, 1H), 7.76 (td, *J* = 1.2, 8.0 Hz, 1H), 7.69 (td, *J* = 1.2, 8.0 Hz, 1H), 7.56 (d, *J* = 8.0 Hz, 1H), 2.68 (s, 3H). ^13^C-NMR: δ ppm 153.3, 144.6, 141.6, 132.7, 130.0, 129.3, 128.7, 128.6, 126.8, 124.6, 124.0, 122.2, 121.5, 22.5. HRMS (ESI^+^): calcd 194.0964 for C_14_H_12_N^+^ [M + H]^+^; found, 194.0969.

*3-Methoxy-9-methylphenanthridine* (**4r**): White amorphous powder, ^1^H-NMR: δ ppm 9.21 (s, 1H), 8.46 (d, *J* = 8.8 Hz, 1H), 8.30 (s, 1H), 7.92 (d, *J* = 8.0 Hz, 1H), 7.60 (d, *J* = 2.4 Hz, 1H), 7.47 (d, *J* = 8.0 Hz, 1H), 7.31 (dd, *J* = 2.4, 8.8 Hz, 1H), 4.01 (s, 3H), 2.65 (s, 3H). ^13^C-NMR (100 MHz, CDCl_3_): δ ppm 160.0, 153.6, 146.2, 141.6, 133.0, 128.7, 128.2, 123.8, 123.4, 121.0, 118.0, 117.9, 109.8, 55.6, 22.5. HRMS (ESI^+^): calcd 224.1070 for C_15_H_14_NO^+^ [M + H]^+^; found, 224.1066.

*1-Methylphenanthridine* (**7a**): White amorphous powder, ^1^H-NMR: δ ppm 8.59 (d, *J* = 8.3 Hz, 1H), 8.51 (dd, *J* = 8.2, 1.2 Hz, 1H), 8.19 (dd, *J* = 8.2, 0.6 Hz, 1H), 8.10 (dd, *J* = 8.2, 1.0 Hz, 1H), 7.81 (dd, *J* = 8.2, 7.2 Hz, 1H), 7.73–7.64 (m, 2H), 7.60 (dd, *J* = 8.2, 7.2 Hz, 1H), 3.03 (s, 3H). ^13^C-NMR: δ ppm 158.9, 143.6, 132.6, 130.5, 129.3, 128.7, 127.3, 126.6, 126.4, 125.90, 123.8, 122.3, 122.0, 23.4. HRMS (ESI^+^): calcd 194.0964 for C_14_H_12_N^+^ [M + H]^+^; found, 194.09604.

*3,6-Dimethylphenanthridine* (**7b**): White amorphous powder, ^1^H-NMR: δ ppm 8.54 (d, *J* = 8.0 Hz, 1H), 8.38 (d, *J* = 8.4 Hz, 1H), 8.16 (dd, *J* = 0.8, 8.4 Hz, 1H), 7.91 (s, 1H), 7.79 (td, *J* = 1.6, 8.0 Hz, 1H), 7.63 (td, *J* = 1.2, 8.0 Hz, 1H), 7.43 (dd, *J* = 1.6, 8.4 Hz, 1H), 3.02 (s, 3H), 2.59 (s, 3H). ^13^C-NMR: δ ppm 158.8, 143.8, 138.7, 132.6, 130.4, 129.0, 128.0, 126.8, 126.5, 125.6, 122.1, 121.7, 121.4, 23.3, 21.6. HRMS (ESI^+^): calcd 208.1121 for C_15_H_14_N^+^ [M + H]^+^; found, 208.1124.

*3-Methoxy-6-methylphenanthridine* (**7c**): Pale yellow amorphous powder, ^1^H-NMR: δ ppm 8.44 (d, *J* = 8.4 Hz, 1H), 8.35 (d, *J* = 9.2 Hz, 1H), 8.13 (dd, *J* = 0.4, 8.0 Hz, 1H), 7.75 (brt, *J* = 8.4 Hz, 1H), 7.56 (td, *J* = 0.8, 8.0 Hz, 1H), 7.51 (d, *J* = 2.8 Hz, 1H), 7.22 (dd, *J* = 2.4, 8.8 Hz, 1H), 3.97 (s, 3H), 3.00 (s, 3H). ^13^C-NMR: δ ppm 160.1, 159.3, 145.2, 132.7, 130.5, 126.5, 126.1, 124.9, 123.1, 121.8, 117.7, 117.2, 109.3, 55.5, 23.2. HRMS (ESI^+^): calcd 224.1070 for C_15_H_14_NO^+^ [M + H]^+^; found, 224.1066.

*3-Fluoro-6-methylphenanthridine* (**7d**): White amorphous powder, ^1^H-NMR: δ ppm 8.30 (d, *J* = 8.4 Hz, 1H), 8.48 (dd, *J* = 7.0, 8.8 Hz, 1H), 8.20 (d, *J* = 8.4 Hz, 1H), 7.83 (td, *J* = 8.0, 0.8 Hz, 1H), 7.74 (dd, *J* = 10.0, 2.8 Hz, 1H), 7.68 (t, *J* = 7.2 Hz, 1H), 7.36 (td, *J* = 2.8, 8.8 Hz, 1H), 3.03 (s, 3H). ^13^C-NMR: δ ppm 162.8 (d, ^1^*J*_F-C_ = 246.1 Hz), 160.5, 145.1(d, ^3^*J*_F-C_ = 11.6 Hz), 132.5, 131.0, 127.3, 126.8, 125.6, 124.0 (d, ^3^*J*_F-C_ = 5.0 Hz), 122.2, 120.6, 115.4 (d, ^2^*J*_F-C_ = 23.8 Hz), 114.1 (d, ^2^*J*_F-C_ = 20.3 Hz), 23.5. HRMS (ESI^+^): calcd 212.0870 for C_14_H_11_FN^+^ [M + H]^+^; found, 212.0877.

*3-Chloro-6-methylphenanthridine* (**7e**): White amorphous powder, ^1^H-NMR: δ ppm 8.52 (d, *J* = 8.0 Hz, 1H), 8.41 (d, *J* = 8.4 Hz, 1H), 8.19 (d, *J* = 8.0 Hz, 1H), 8.07 (d, *J* = 2.0 Hz, 1H), 7.82 (td, *J* = 8.0, 1.2 Hz, 1H), 7.70 (td, *J* = 8.0, 1.2 Hz, 1H), 7.54 (dd, *J* = 8.8, 2.4 Hz, 1H), 3.01 (s, 3H). ^13^C-NMR: δ ppm 160.4, 144.5, 134.3, 132.2, 131.0, 128.8, 127.7, 127.0, 126.8, 126.0, 123.4, 122.4, 122.4, 23.5. HRMS (ESI^+^): calcd 228.0575 for C_14_H_11_ClN^+^ [M + H]^+^; found, 228.0570.

*6-Methyl-3-(trifluoromethyl)phenanthridine* (**7f**): Pale yellow amorphous powder, ^1^H-NMR: δ ppm 8.49 (brd, *J* = 7.2 Hz, 2H), 8.34 (brs, 1H), 8.17–8.16 (m, 1H), 7.82 (brd, *J* = 7.2 Hz, 1H), 7.73 (brs, 2H), 3.00 (brs, 3H). ^13^C-NMR: δ ppm 160.4, 142.9, 131.5, 130.9, 130.4, 130.1, 128.4, 126.8, 126.7, 126.5, 126.1 (q ^1^*J*_F-C_ = 257.0 Hz), 122.8, 122.5, 122.0, 23.3. HRMS (ESI^+^): calcd 262.0838 for C_15_H_11_F3N^+^ [M + H]^+^; found, 262.0847.

## 4. Conclusions

In summary, a metal-free visible-light photoredox catalyzed intermolecular cyclization reaction of *O*-2,4-dinitrophenyl oximes to phenanthridines was developed in this study. Compared with Ru or Ir complexes, the organic dye type photocatalyst eosin Y used in this research is much cheaper. Furthermore, the reaction conditions for cyclization of *O*-2,4-dinitrophenyl oximes in this study were simple, mild and environmentally-friendly. Future studies will focus on applying this method to the synthesis other *N*-containing heterocycles based on tandem radical reactions.

## Figures and Tables

**Table 1:**
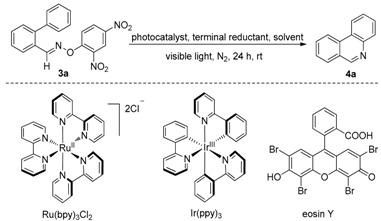
Optimization for the photoredox catalyzed intramolecular cyclization of **3a** ^a^.

Entry	Photocatalyst	Terminal Reductant	Solvent	Yield of 4a ^b^
1	Ru(bpy)_3_C_l2_·6H_2_O	-	MeCN	6%
2	Ru(bpy)_3_C_l2_·6H_2_O	*i*-Pr_2_NEt	MeCN	32%
3	Ru(bpy)_3_C_l2_·6H_2_O	*i*-Pr_2_NEt	DMSO	37%
4	Ru(bpy)_3_C_l2_·6H_2_O	*i*-Pr_2_NEt	DMF	46%
5	Ir(ppy)_3_	-	DMF	28%
6	Ir(ppy)_3_	*i*-Pr_2_NE	DMF	51%
7	eosin Y	-	DMF	75%
8	eosin Y	*i*-Pr_2_NEt	DMF	74% ^c^
9	eosin Y	*i*-Pr_2_NEt	DMF	0% ^d^
10	-	*i*-Pr_2_NEt	DMF	7%
11	-	*i*-Pr_2_NEt	DMF	0% ^d^
12	-	-	DMF	trace ^e^

^a^ Reaction conditions: **3a** (0.2 mmol), photocatalyst (2 mol %), terminal reductant (3.0 equiv.), solvent (2.0 mL), 25 W compact fluorescent light bulb, under nitrogen atmosphere for 24 h; ^b^ Isolated yields; ^c^ 12 h reaction; ^d^ reaction was carried out in darkness; ^e^ 100 °C for 6 h.

**Table 2:**
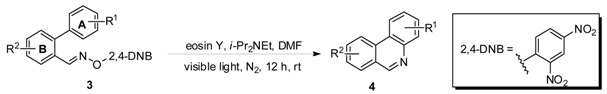
Scope of substituent group on aryl ring of *O*-2,4-dinitrophenyl oximes **3** ^a^.

Substrate	Product	Substrate	Product
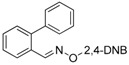	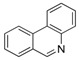	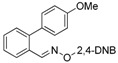	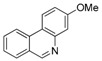
**3a**	**4a** 74%	**3b**	**4b** 51%
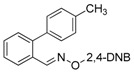	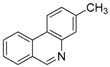	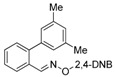	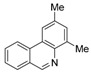
**3c**	**4c** 46%	**3d**	**4d** 49%
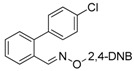	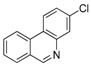	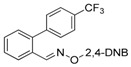	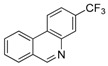
**3e**	**4e** 53%	**3f**	**4f** 80%
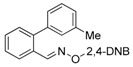	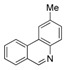 + 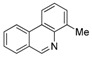	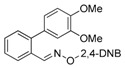	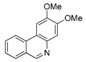 + 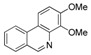
**3g**	**4ga** 33% **4gb** 17%	**3h**	**4ha** 38% **4hb** 20%
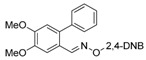	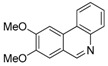	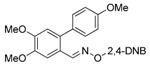	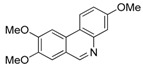
**3i**	**4i** 51%	**3j**	**4j** 46%
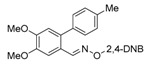	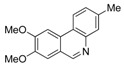	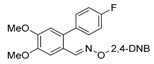	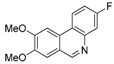
**3k**	**4k** 40%	**3l**	**4l** 49%
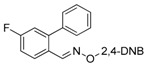	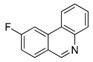	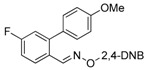	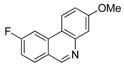
**3m**	**4m** 53%	**3n**	**4n** 46%
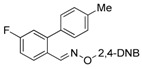	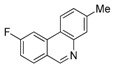	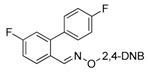	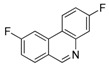
**3o**	**4o** 43%	**3p**	**4p** 57%
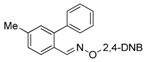	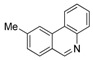	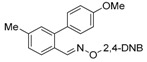	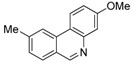
**3q**	**4q** 49%	**3r**	**4r** 43%

^a^ Reaction conditions: **3** (0.5 mmol), photocatalyst (2 mol %), *i*-Pr_2_NEt (3.0 equiv.), solvent (5.0 mL), 25 W compact fluorescent light bulb, under nitrogen atmosphere for 12 h.

**Table 3:**
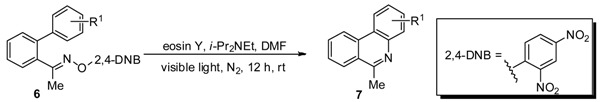
Cyclization of *O*-2,4-dinitrophenyl acetophenone oximes **6** to phenanthridines **7** ^a^.

Substrate	Product	Substrate	Product
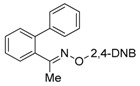	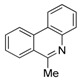	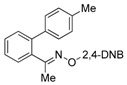	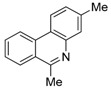
**6a**	**7a** 92%	**6b**	**7b** 89%
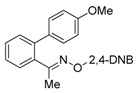	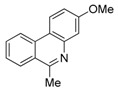	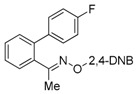	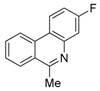
**6c**	**7c** 88%	**7d**	**7d** 91%
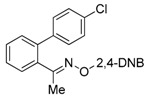	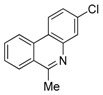	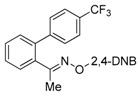	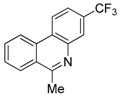
**6e**	**7e** 85%	**6f**	**7f** 90%

^a^ Reaction conditions: **3** (0.5 mmol), photocatalyst (2 mol %), *i*-Pr_2_NEt (3.0 equiv.), solvent (5.0 mL), 25 W compact fluorescent light bulb, under nitrogen atmosphere for 12 h.

## Supplementary Materials

Supplementary materials can be accessed at: http://www.mdpi.com/1420-3049/21/12/1690/s1.

## References

[1] Zenk M.H. (1994). The formation of benzophenanthridine alkaloids. Pure Appl. Chem..

[2] Nakanishi T., Suzuki M. (1998). Revision of the structure of fagaridine based on the comparison of UV and NMR data of synthetic compounds. J. Nat. Prod..

[3] Nakanishi T., Masuda A., Suwa M., Akiyama Y., Hoshino-Abe N., Suzuki M. (2000). Synthesis of derivatives of NK109, 7-OH benzo[*c*]phenanthridine alkaloid, and evaluation of their cytotoxicities and reduction-resistant properties. Bioorg. Med. Chem. Lett..

[4] Nakanishi T., Suzuki M., Mashiba A., Ishikawa K., Yokotsuka T. (1998). Synthesis of NK109, an anticancer benzo[*c*]phenanthridine alkaloid. J. Org. Chem..

[5] Newman S.E., Roll M.J., Harkrader R.J. (1999). A naturally occurring compound for controlling powdery mildew of greenhouse roses. HortScience.

[6] Seaman A., Woodbine M. (1954). The antibacterial activity of phenanthridine compounds. Brit. J. Pharmacol..

[7] Schrader K.K., Avolio F., Andolfi A., Cimmino A., Evidente A. (2013). Ungeremine and its hemisynthesized analogues as bactericides against *Flavobacterium columnare*. J. Agric. Food Chem..

[8] Kellinger M.W., Park G.Y., Chong J., Lippard S.J., Wang D. (2013). Effect of a monofunctional phenanthriplatin-DNA adduct on RNA polymerase II transcriptional fidelity and translesion synthesis. J. Am. Chem. Soc..

[9] Johnstone T.C., Alexander S.M., Lin W., Lippard S.J. (2014). Effects of monofunctional platinum agents on bacterial growth: A retrospective study. J. Am. Chem. Soc..

[10] Baechler S.A., Fehr M., Habermeyer M., Hofmann A., Merz K., Fiebig H., Marko D., Eisenbrand G. (2013). Synthesis, topoisomerase-targeting activity and growth inhibition of lycobetaine analogs. Bioorg. Med. Chem..

[11] Cheng P., Zhou J., Qing Z., Kang W., Liu S., Liu W., Xie H., Zeng J. (2014). Synthesis of 5-methyl phenanthridium derivatives: A new class of human DOPA decarboxylase inhibitors. Bioorg. Med. Chem. Lett..

[12] Stevens N., O’Connor N., Vishwasrao H., Samaroo D., Kandel E.R., Akins D.L., Drain C.M., Turro N. (2008). Two color RNA intercalating probe for cell imaging applications. J. Am. Chem. Soc..

[13] Candito D.A., Lautens M. (2009). Palladium-catalyzed domino direct arylation/N-arylation: Convenient synthesis of phenanthridines. Angew. Chem. Int. Ed..

[14] Blanchot M., Candito D.A., Larnaud F., Lautens M. (2011). Formal synthesis of nitidine and NK109 via palladium-catalyzed domino direct arylation/N-arylation of aryl triflates. Org. Lett..

[15] Zhang B., Studer A. (2015). Recent advances in the synthesis of nitrogen heterocycles via radical cascade reactions using isonitriles as radical acceptors. Chem. Soc. Rev..

[16] Zhang B., Mück-Lichtenfeld C., Daniliuc C.G., Studer A. (2013). 6-Trifluoromethylphenanthridines through radical trifluoromethylation of isonitriles. Angew. Chem. Int. Ed..

[17] Zhang B., Studer A. (2014). 2-Trifluoromethylated indoles via radical trifluoromethylation of isonitriles. Org. Lett..

[18] Zhang B., Studer A. (2014). 6-Perfluoroalkylated phenanthridines via radical perfluoroalkylation of isonitriles. Org. Lett..

[19] Rortela-Cubillo F., Scott J.S., Walton J.C. (2007). Microwave-assisted preparations of dihydropyrroles from alkenone O-phenyl oximes. Chem. Commun..

[20] Cubillo F., Scanlan E.M., Scott J.S., Walton J.C. (2008). From dioxime oxalates to dihydropyrroles and phenanthridines via iminyl radicals. Chem. Commun..

[21] Cubillo F., Scott J.S., Walton J.C. (2008). Microwave-assisted syntheses of *N*-heterocycles using alkenone-, alkynone- and aryl-carbonyl *O*-phenyl oximes: Formal synthesis of neocryptolepine. J. Org. Chem..

[22] McBurney R.T., Walton J.C. (2013). Dissociation or cyclization: Options for a triad of radicals released from oxime carbamates. J. Am. Chem. Soc..

[23] Walton J.C. (2014). The oxime portmanteau motif: Released heteroradicals undergo incisive EPR interrogation and deliver diverse heterocycles. Acc. Chem. Res..

[24] Chen J.-R., Hu X.-Q., Lu L.-Q., Xiao W.-J. (2016). Visible light photoredox-controlled reactions of *N*-radicals and radical ions. Chem. Soc. Rev..

[25] Jiang H., An X., Tong K., Zheng T., Zhang Y., Yu S. (2015). Visible-light-promoted iminyl-radical formation from acyl oximes: A unified approach to pyridines, quinolines, and phenanthridines. Angew. Chem. Int. Ed..

[26] An X.-D., Yu S. (2015). Visible-light-promoted and one-pot synthesis of phenanthridines and quinolines from aldehydes and *O*-acyl hydroxylamine. Org. Lett..

[27] Davies J., Booth S.G., Essafi S., Dryfe R.A.W., Leonori D. (2015). Visible-light-mediated generation of nitrogen-centered radicals: Metal-free hydroimination and iminohydroxylation cyclization reactions. Angew. Chem. Int. Ed..

[28] Davies J., Svejstrup T.D., Reina D.F., Sheikh N.S., Leonori D. (2016). Visible-light-mediated synthesis of amidyl radicals: Transition-metal-free hydroamination and *N*-arylation reactions. J. Am. Chem. Soc..

[29] Cheng P., Zeng J. (2012). Progresses in synthesis of benzophenanthridine alkaloids and their derivatives. Chin. J. Org. Chem..

[30] Zeng J., Liu Y., Liu W., Liu X., Liu F., Huang P., Zhu P., Chen J., Shi M., Guo F. (2013). Integration of transcriptome, proteome and metabolism data reveals the alkaloids biosynthesis in *Macleaya cordata* and *Macleaya microcarpa*. PLoS ONE.

[31] Qing Z.-X., Cheng P., Liu X.-B., Liu Y.-S., Zeng J.-G., Wang W. (2014). Structural speculation and identification of alkaloids in *Macleaya cordata* fruits by high-performance liquid chromatography/quadrupole- time-of-flight mass spectrometry combined with a screening procedure. Rapid Commun. Mass Spectrom..

[32] Qing Z.-X., Liu X.-B., Wu H.-M., Cheng P., Liu Y.-S., Zeng J.-G. (2015). An improved separation method for classification of *Macleaya cordata* from different geographical origins. Anal. Methods.

[33] Xie H., Yang J., Feng S., Cheng P., Zeng J., Xiong X. (2015). Simultaneous quantitative determination of sanguinarine, chelerythrine, dihydrosanguinarine and dihydrochelerythrine in chicken by HPLC-MS/MS method and its applications to drug residue and pharmacokinetic study. J. Chromatogr. B.

[34] Qing Z.-X., Cheng P., Liu X.-B., Liu Y.-S., Zeng J.-G. (2015). Systematic identification of alkaloids in *Macleaya microcarpa* fruits by liquid chromatography tandem mass spectrometry combined with the isoquinoline alkaloids biosynthetic pathway. J. Pharm. Biomed. Anal..

[35] Cheng P., Qing Z., Liu S., Liu W., Xie H., Zeng J. (2014). Regiospecific Minisci acylation of phenanthridine via thermolysis or photolysis. Tetrahedron Lett..

[36] Liu Z., Huang Y., Xie H., Liu W., Zeng J., Cheng P. (2016). A novel C–C radical–radical coupling reaction promoted by visible light: Facile synthesis of 6-substituted *N*-methyl 5,6-dihydrobenzophenanthridine alkaloids. RSC Adv..

[37] Prier C.K., Rankic D.A., MacMillan D.W.C. (2013). Visible light photoredox catalysis with transition metal complexes: Applications in organic synthesis. Chem. Rev..

