# Supplementary material for: Metal-Free Photoredox Catalyzed Cyclization of O-(2,4-Dinitrophenyl)oximes to Phenanthridines
Source: Molecules. 2016 Dec 8;21(12):1690. doi: 10.3390/molecules21121690 (PMC6273968; doi:10.3390/molecules21121690)
Supplement: Supplementary file 1 [file molecules-21-01690-s001.pdf]

Supplementary Materials: Metal-Free Photoredox Catalyzed Cyclization of *O*-(2,4-Dinitrophenyl)oximes to Phenanthridines

Xiubin Liu, Zhixing Qing, Pi Cheng, Xinyu Zheng, Jianguo Zeng, Hongqi Xie

^{1}H and ^{13}C-NMR Spectroscopy

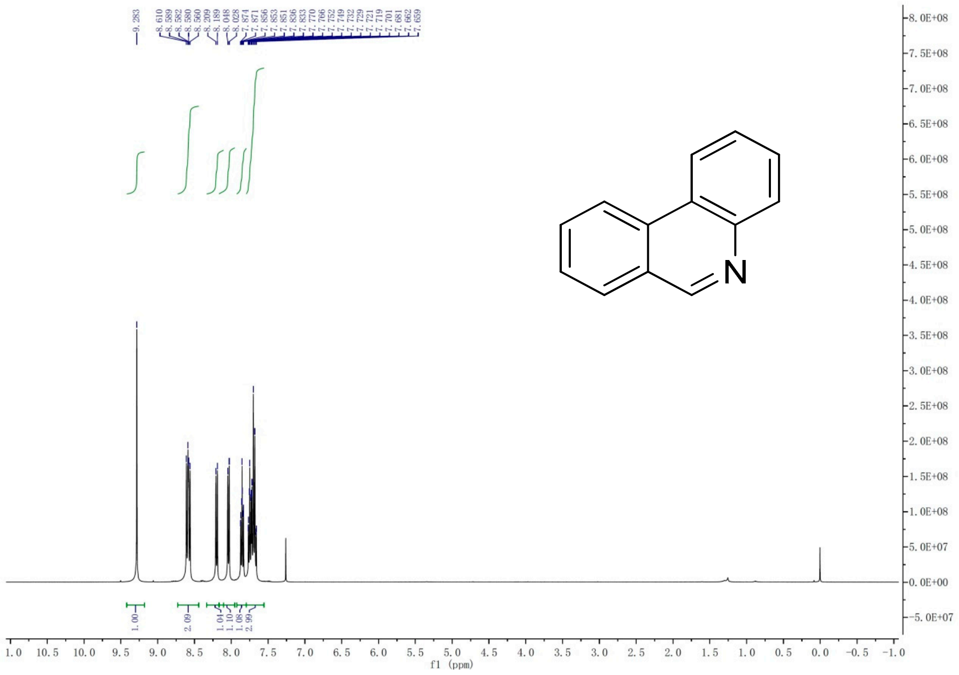

Figure S1. ^{1}H-NMR spectrum of compound **4a**.

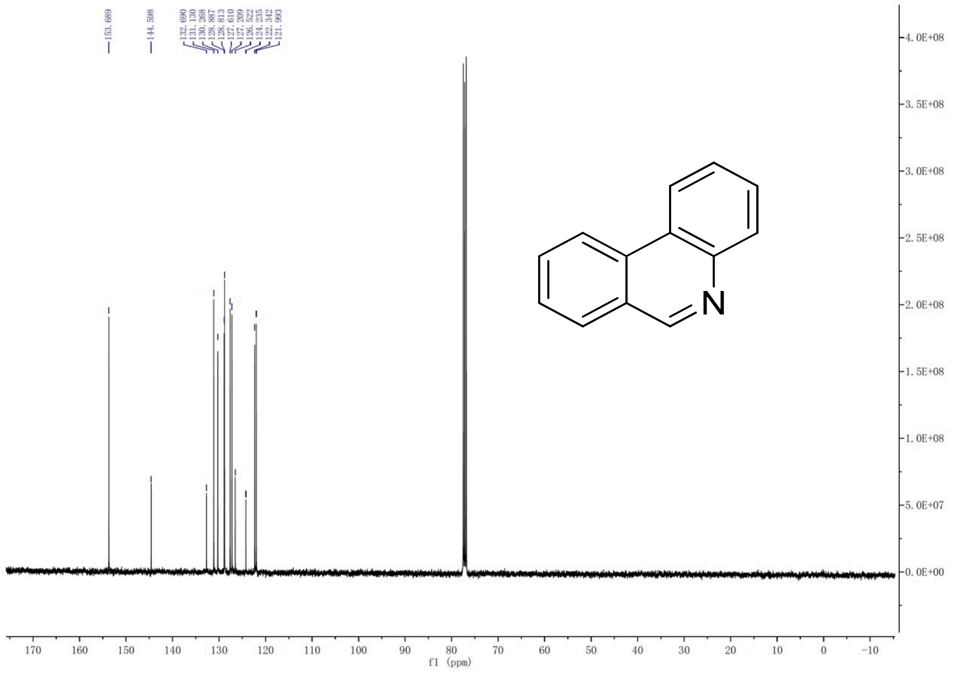

Figure S2. ^{13}C-NMR spectrum of compound name **4a**.

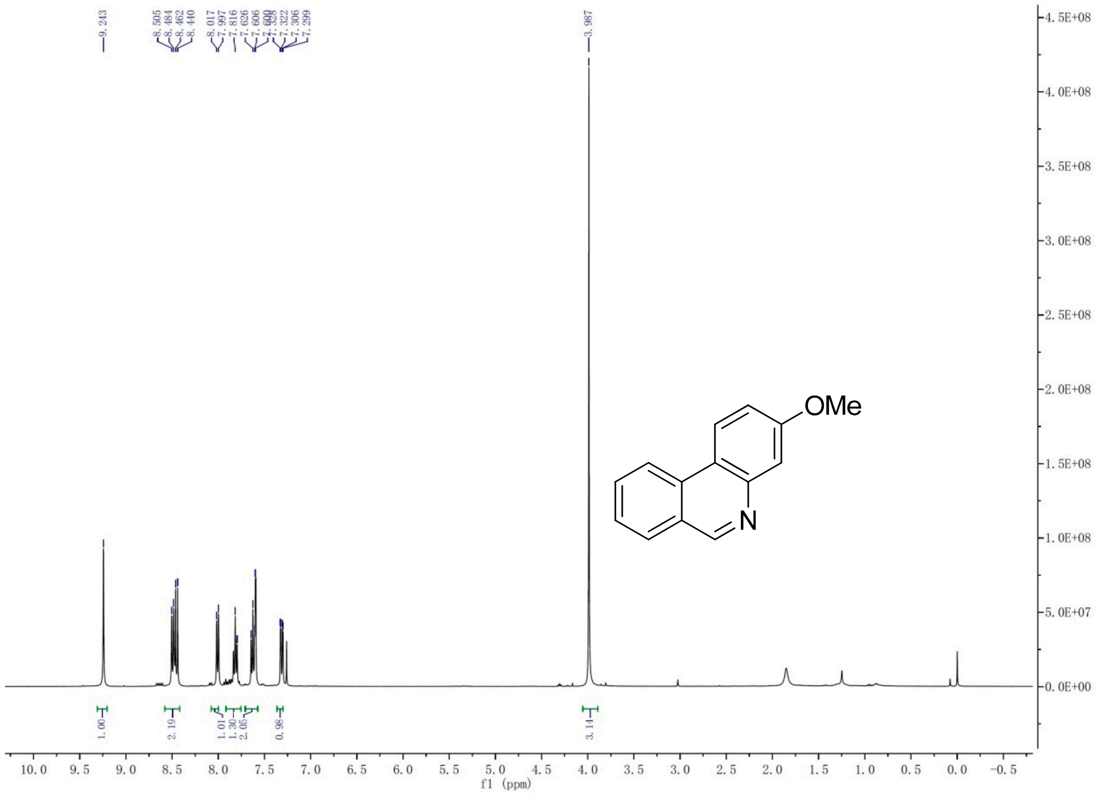

Figure S3. ^{1}H-NMR spectrum of compound **4b**.

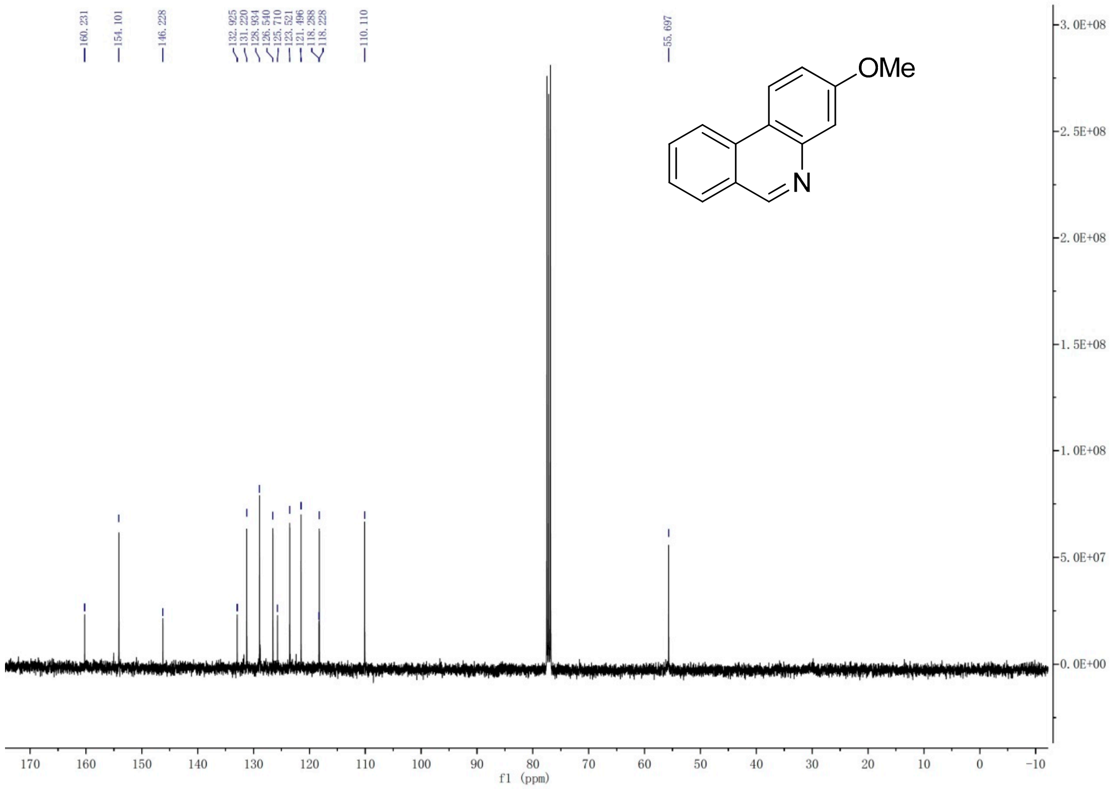

Figure S4. ^{13}C-NMR spectrum of compound name **4b**.

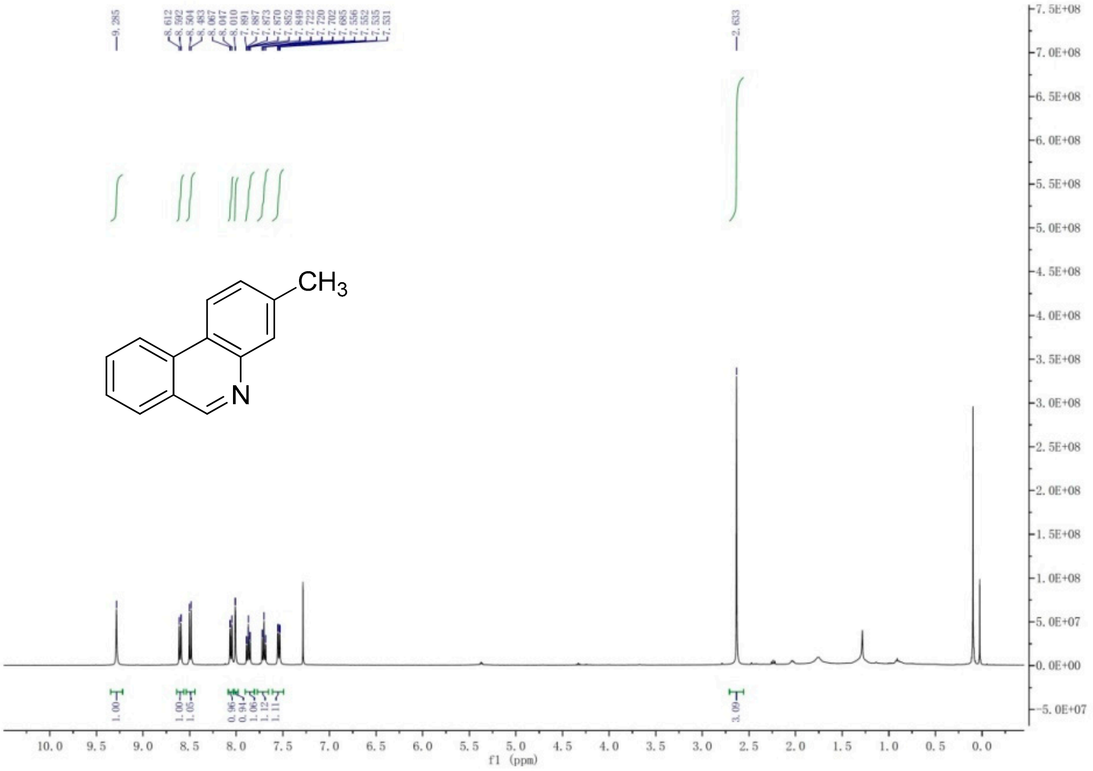

Figure S5. ^{1}H-NMR spectrum of compound **4c**.

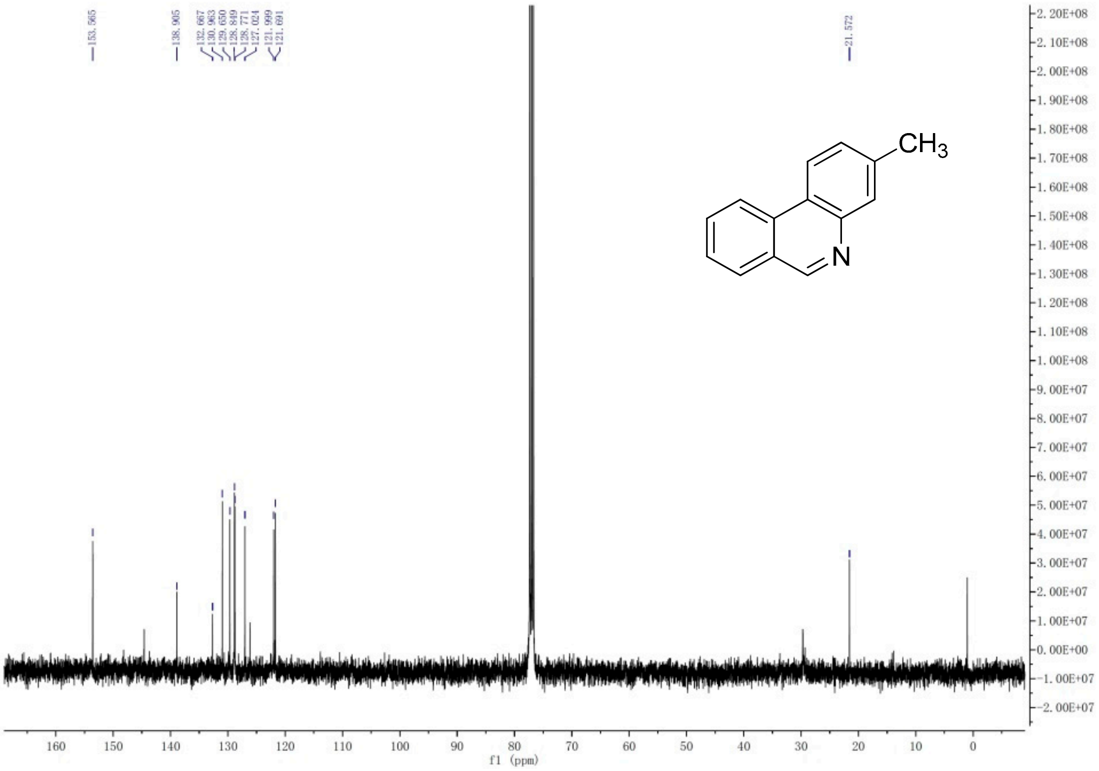

Figure S6. ^{13}C-NMR spectrum of compound name **4c**.

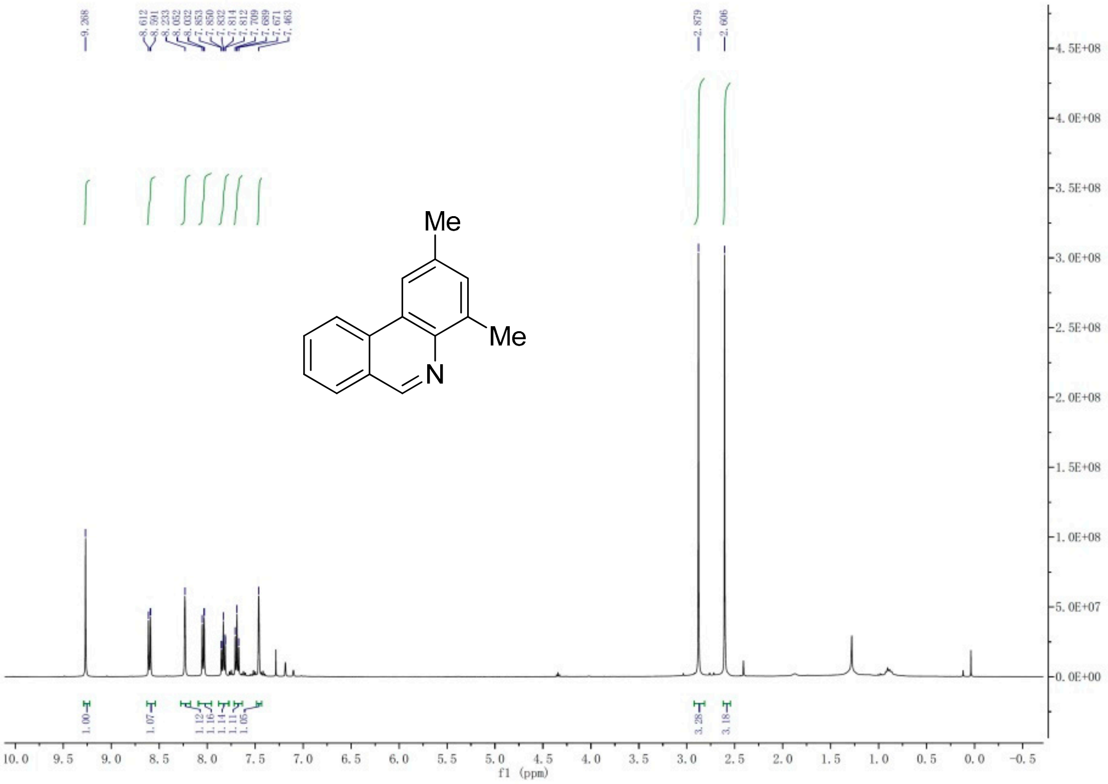

Figure S7. ^{1}H-NMR spectrum of compound **4d**.

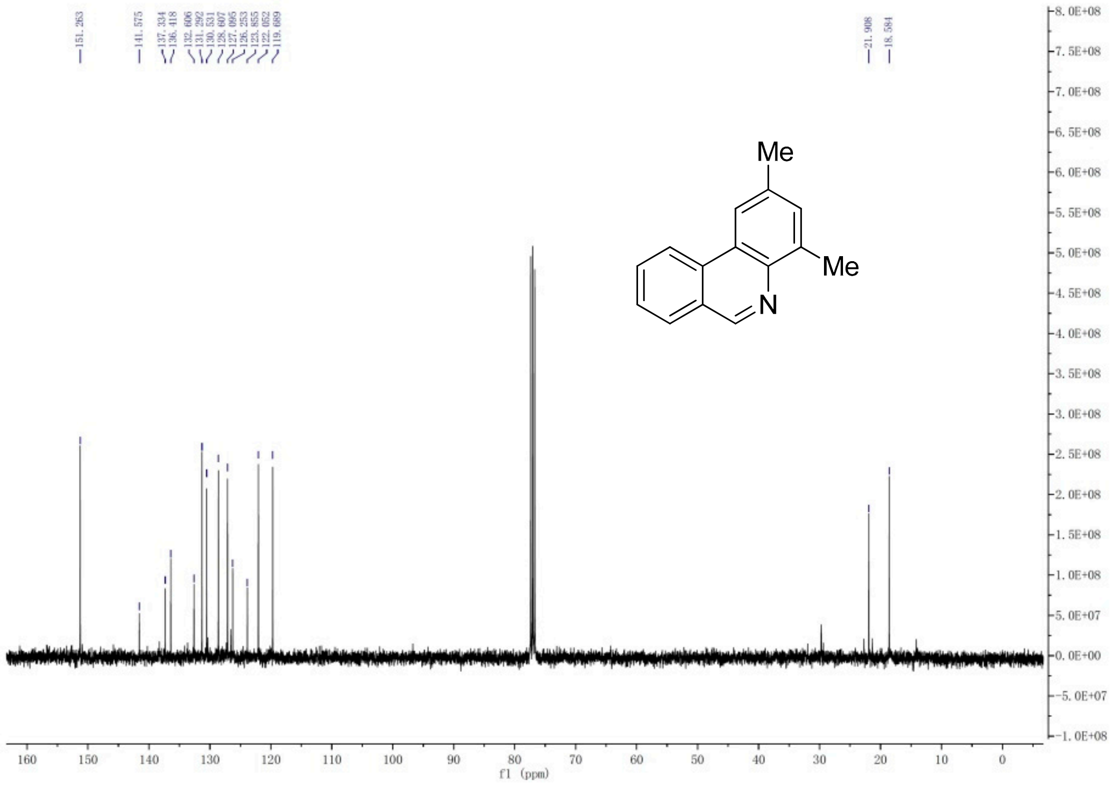

Figure S8. ^{13}C-NMR spectrum of compound name **4d**.

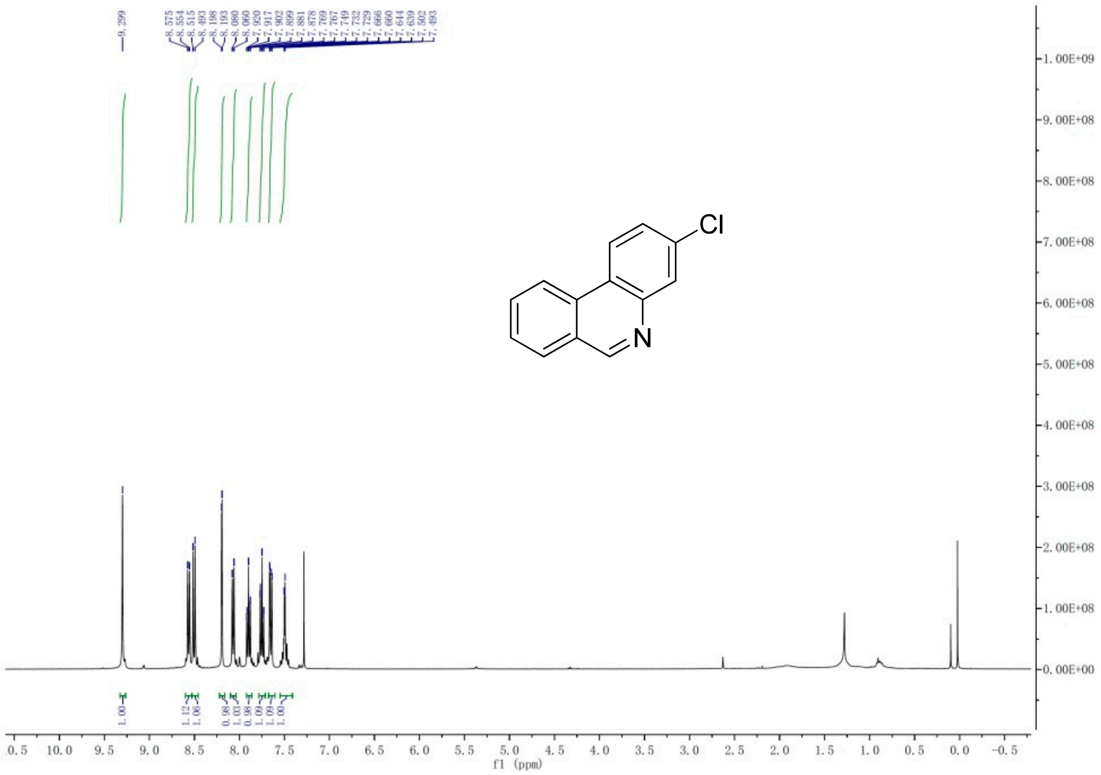

Figure S9. ^{1}H-NMR spectrum of compound **4e**.

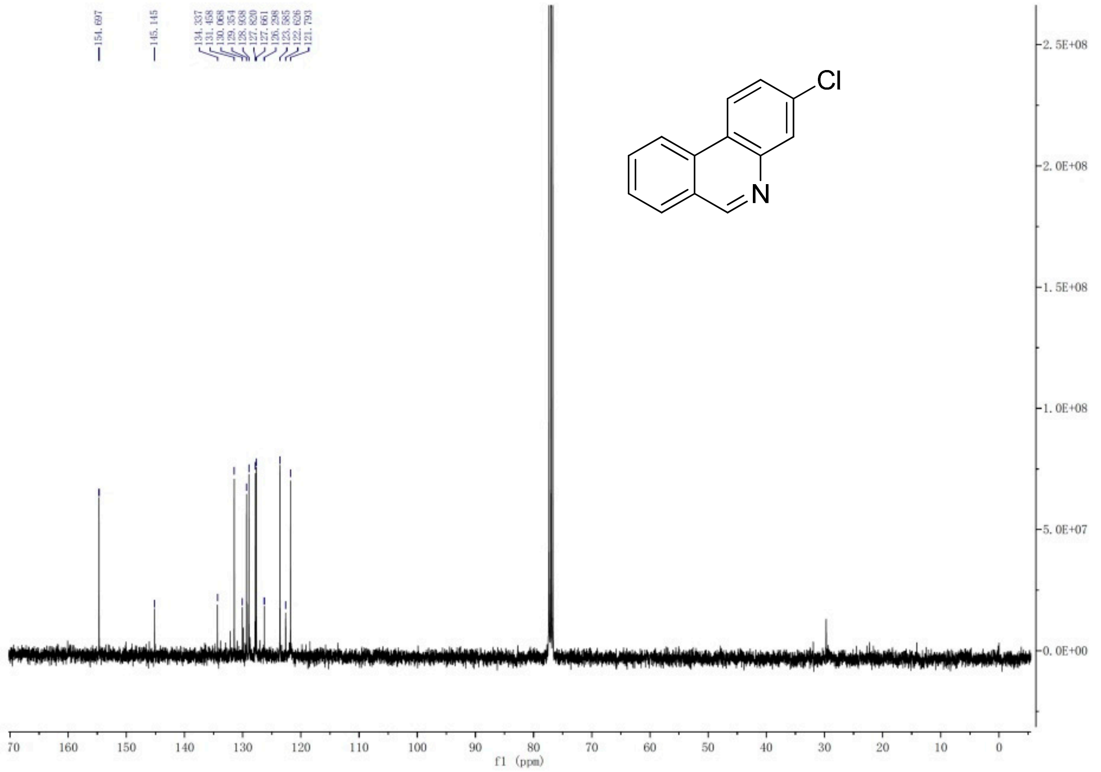

Figure S10. ^{13}C-NMR spectrum of compound name **4e**.

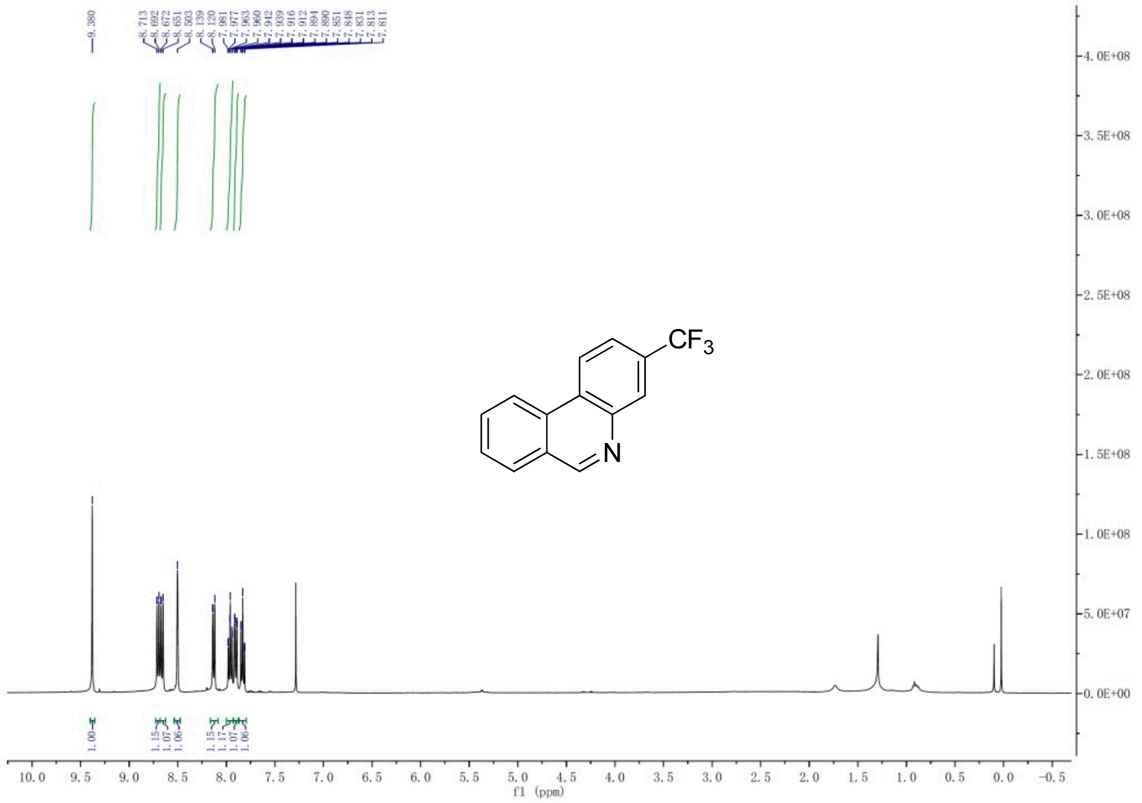

Figure S11. ^{1}H-NMR spectrum of compound **4f**.

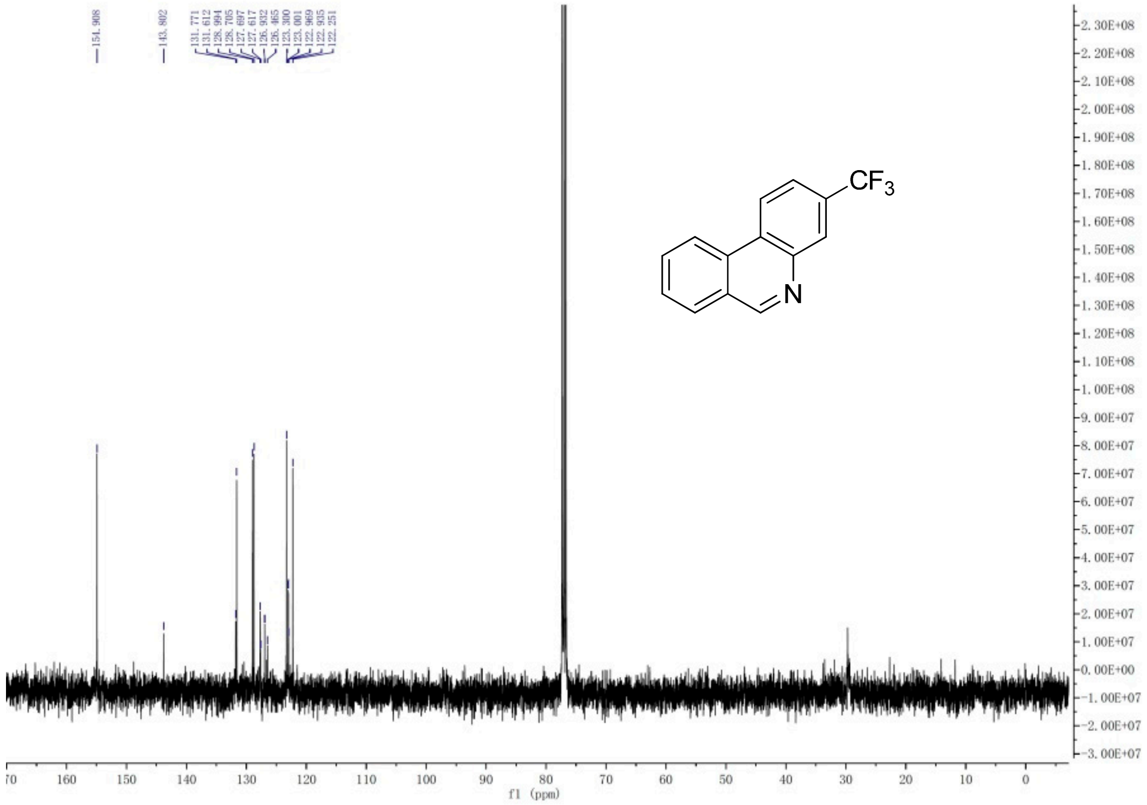

Figure S12 ^{13}C-NMR spectrum of compound name **4f**.

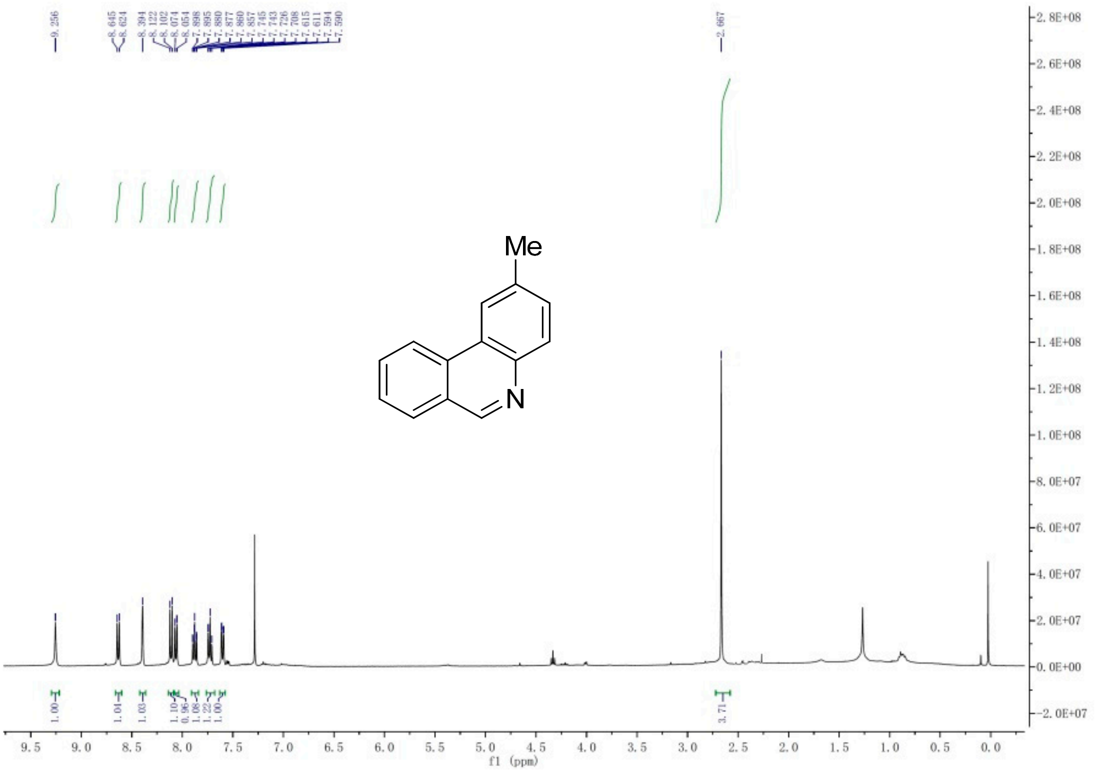

Figure S13. ^{1}H-NMR spectrum of compound **4ga**.

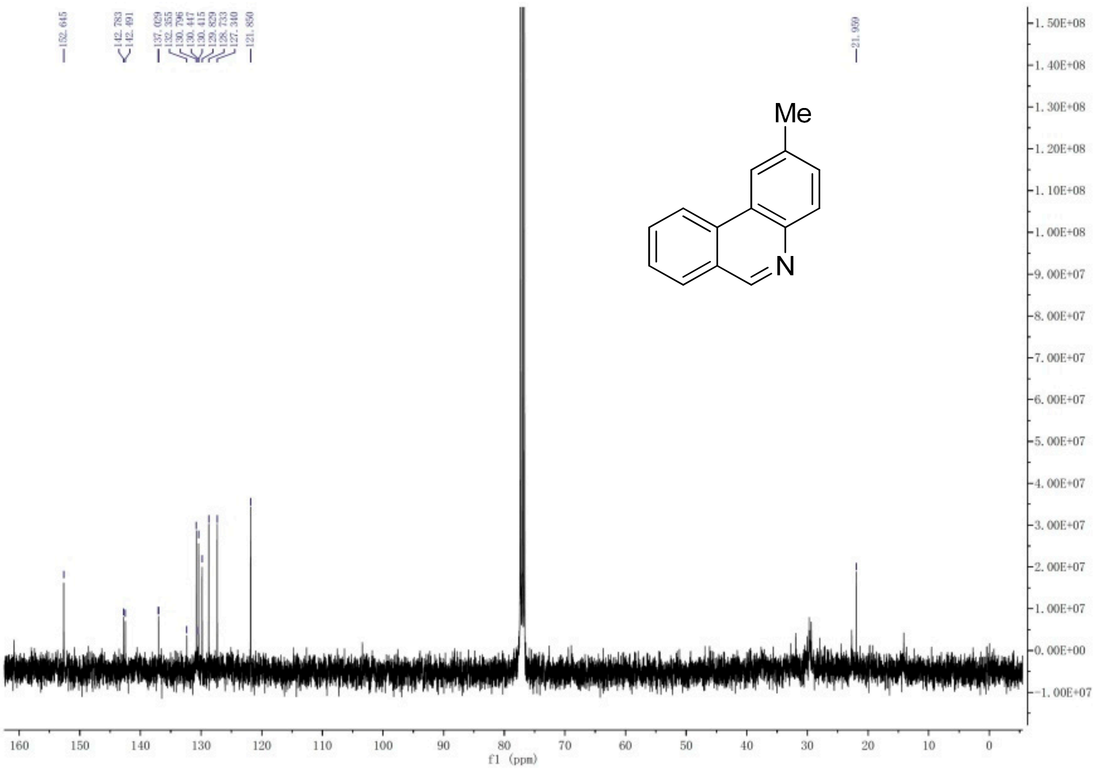

Figure S14. ^{13}C-NMR spectrum of compound **4ga**.

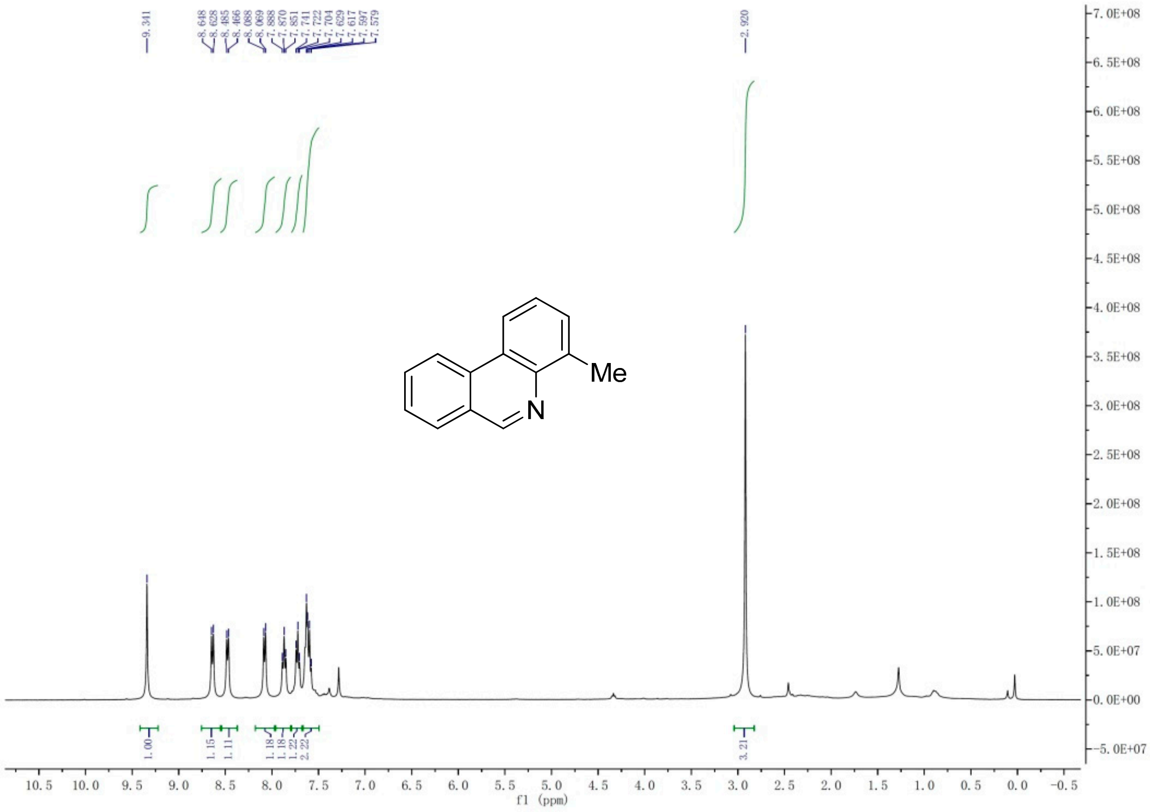

Figure S15. ^{1}H-NMR spectrum of compound **4gb**.

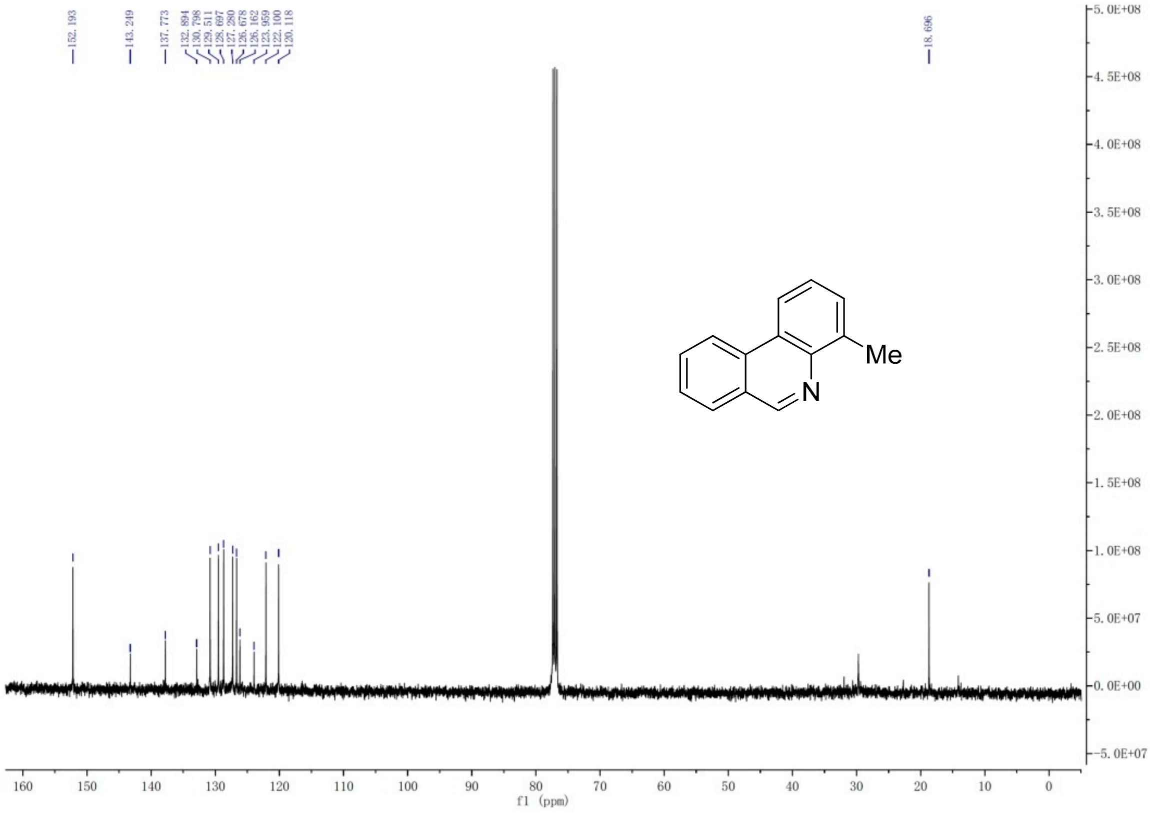

Figure S16. ^{13}C-NMR spectrum of compound **4gb**.

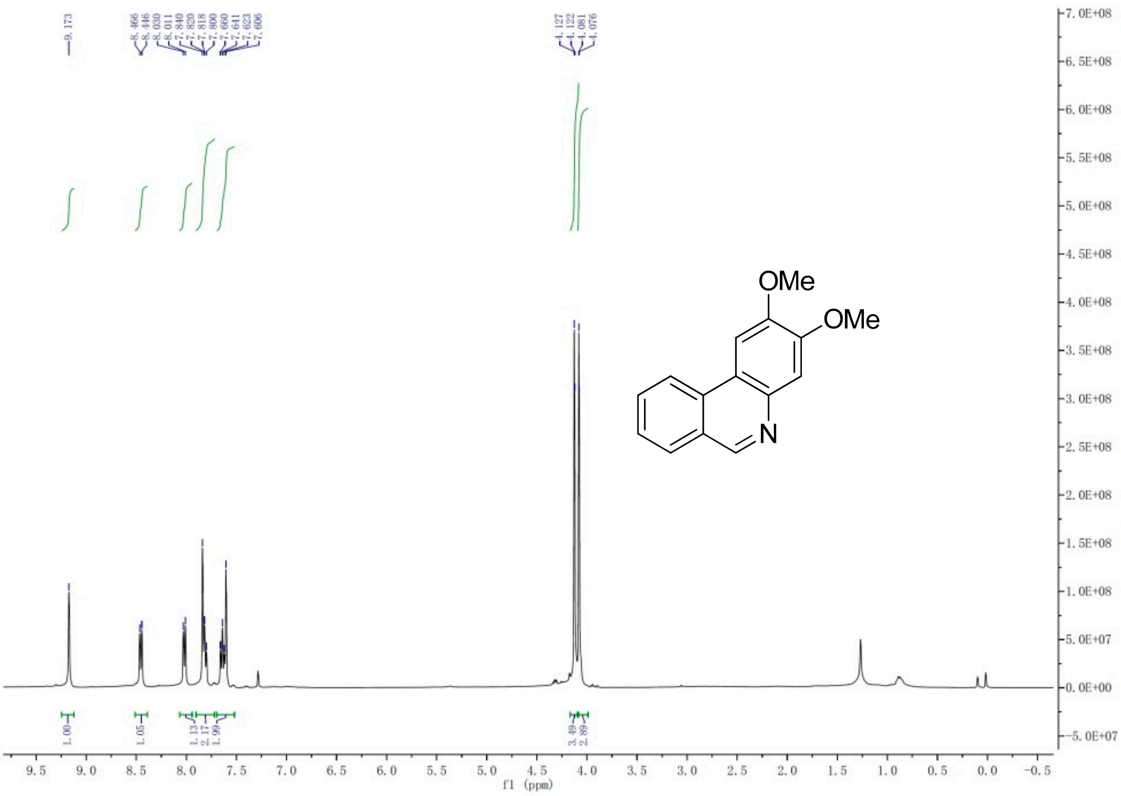

Figure S17. ^{1}H-NMR spectrum of compound **4ha**.

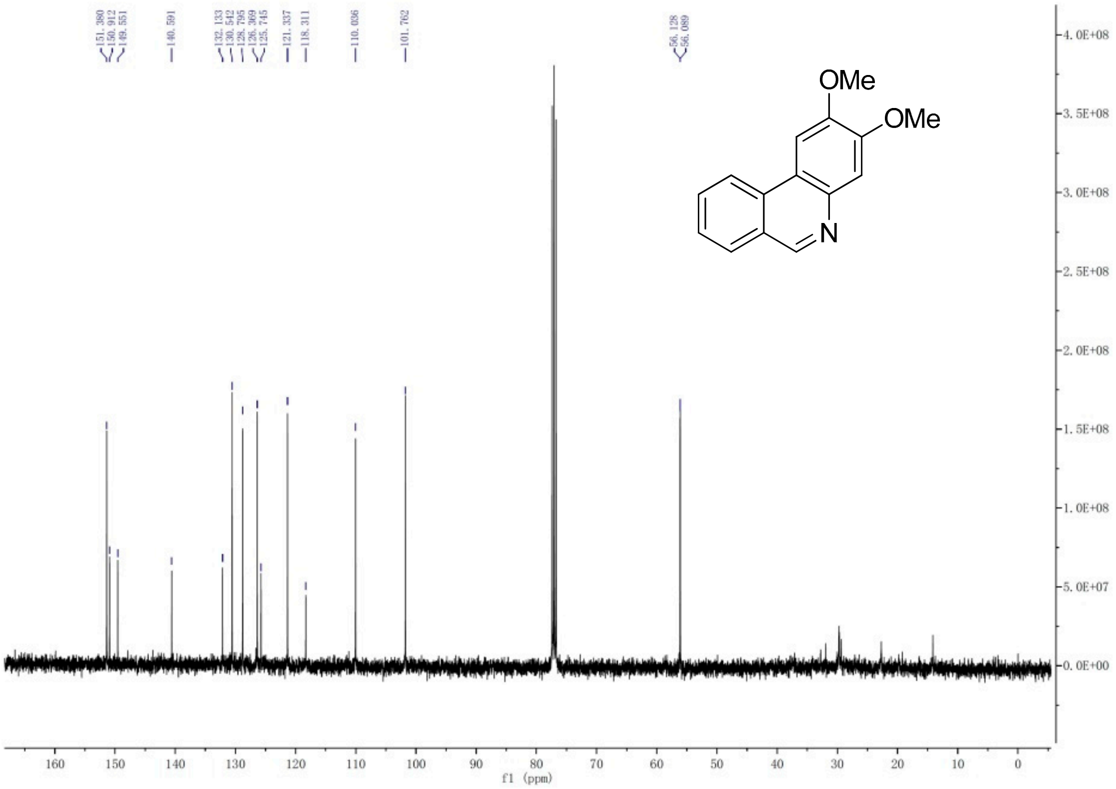

Figure S18. ^{13}C-NMR spectrum of compound **4ha**.

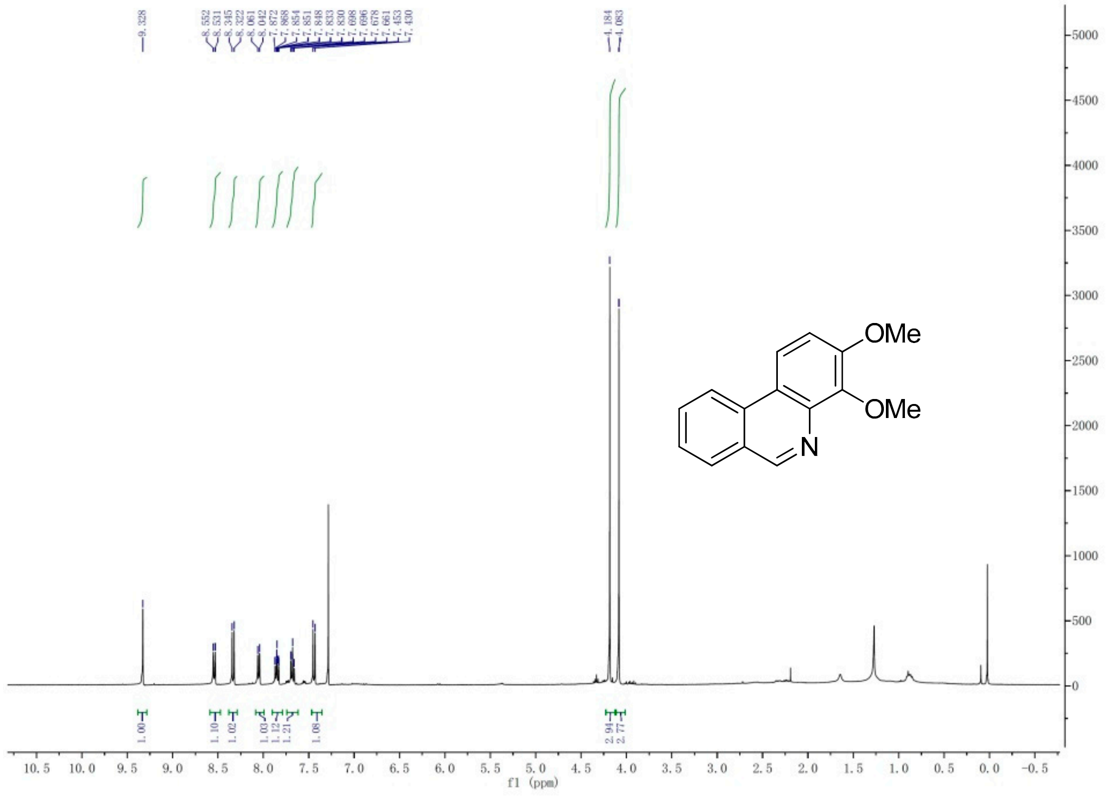

Figure S19. ^{1}H-NMR spectrum of compound **4hb**.

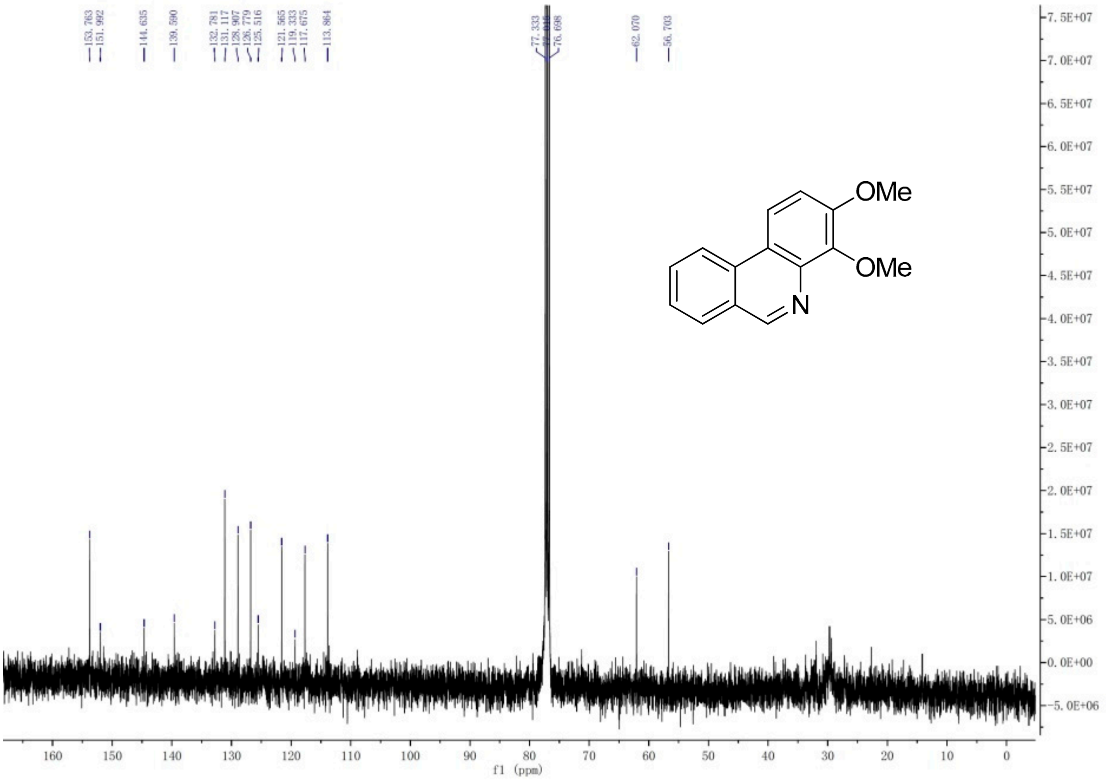

Figure S20. ^{13}C-NMR spectrum of compound **4hb**.

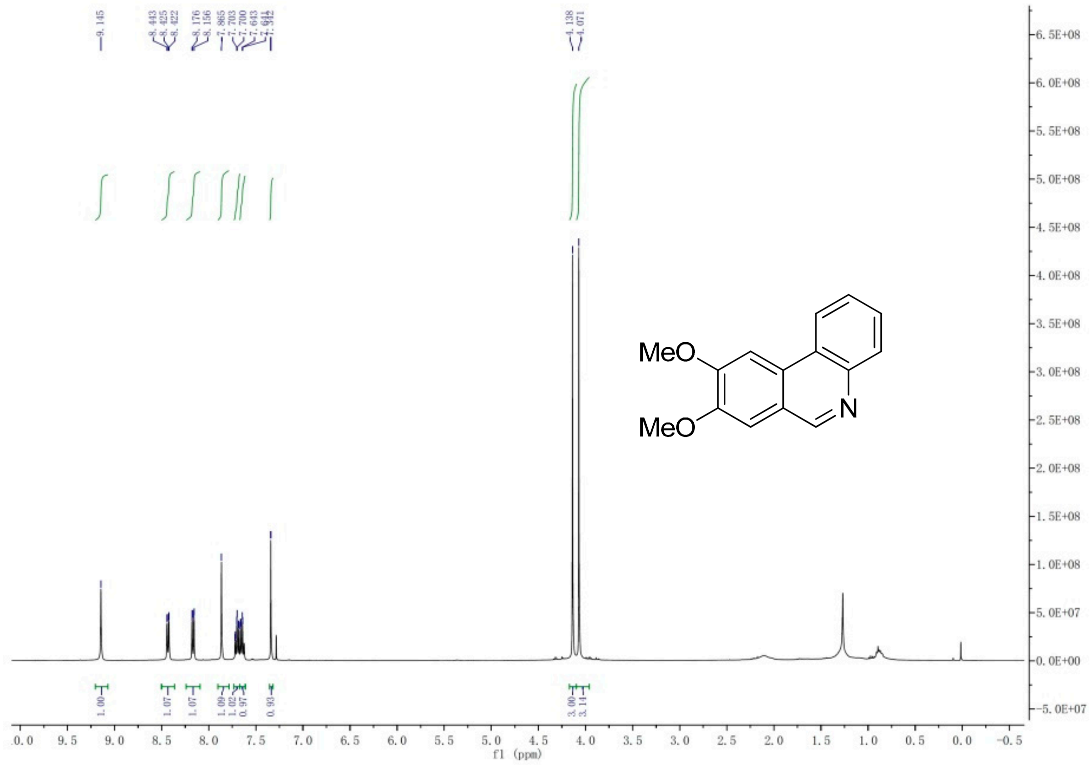

Figure S21. ^{1}H-NMR spectrum of compound **4i**.

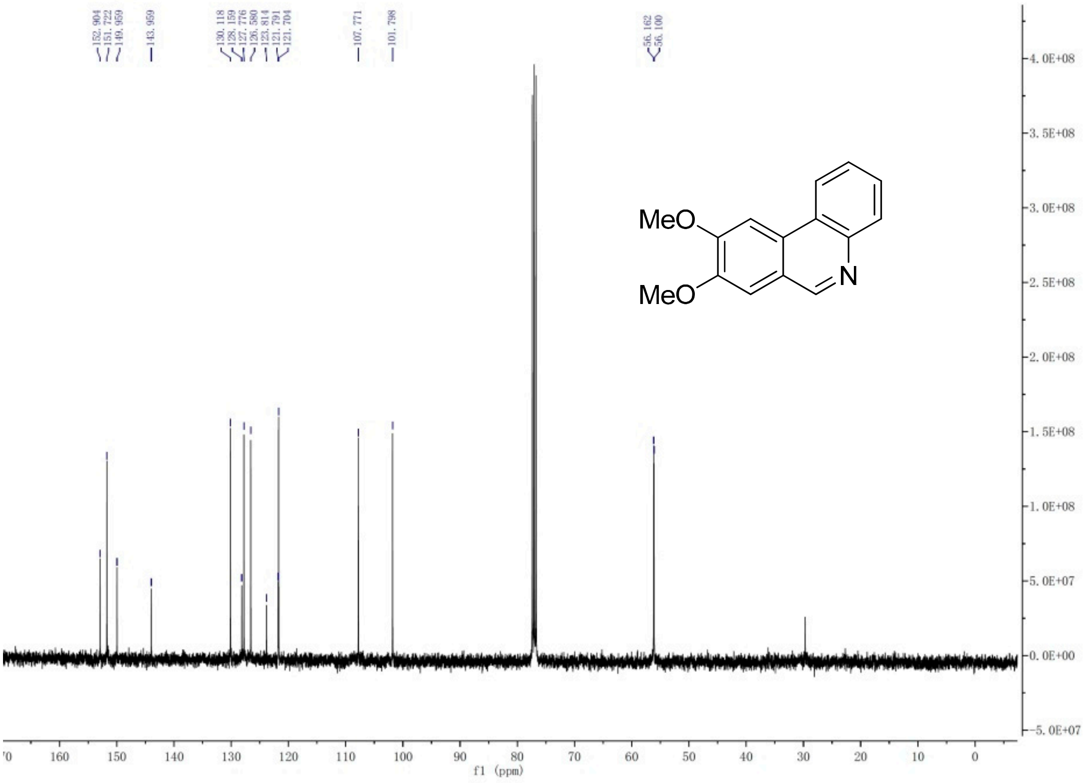

Figure S22. ^{13}C-NMR spectrum of compound **4i**.

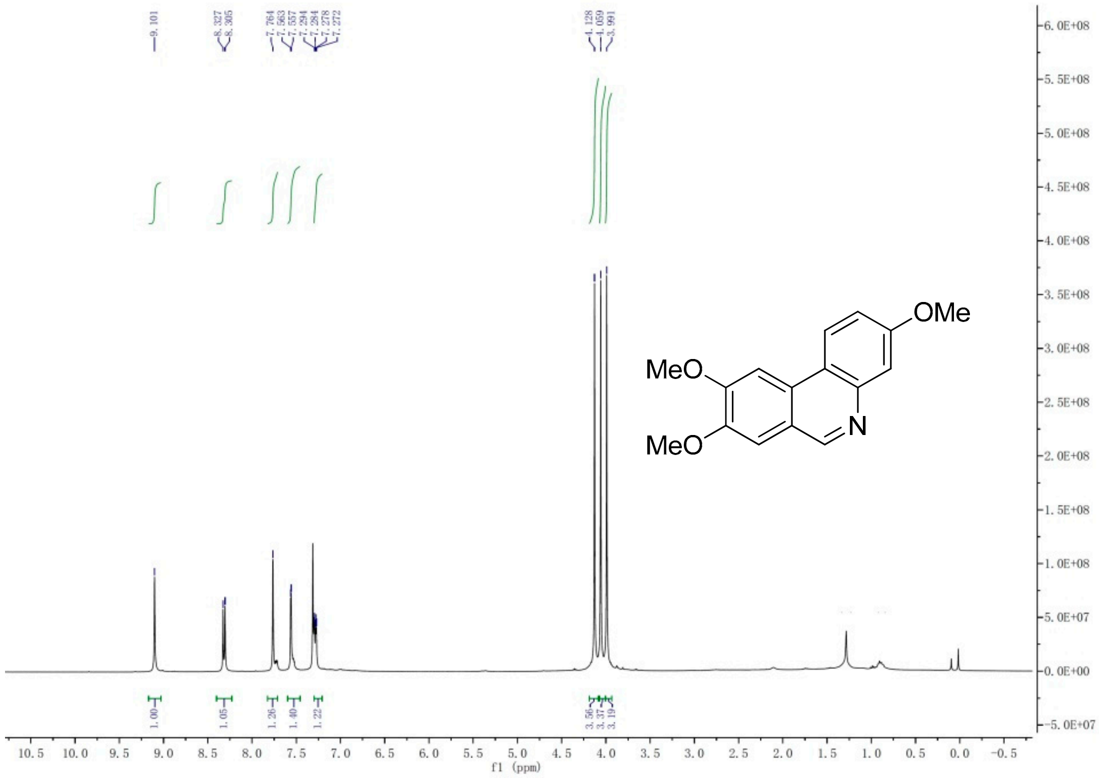

Figure S23. ^{1}H-NMR spectrum of compound **4j**.

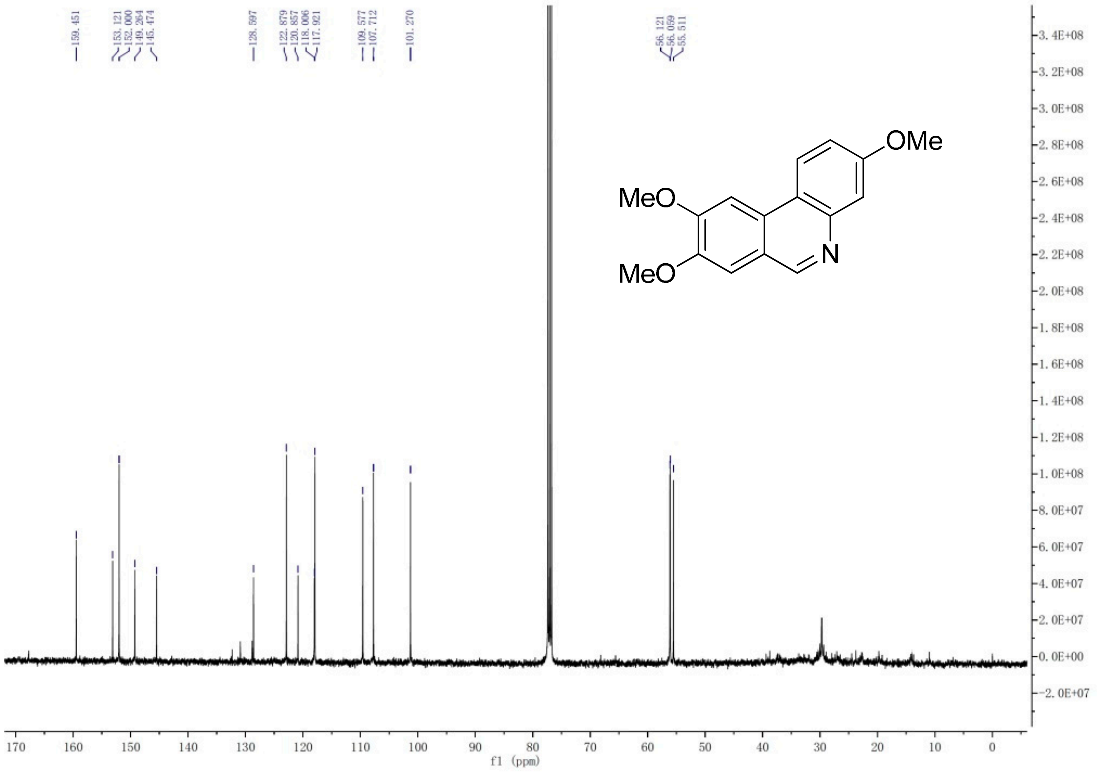

Figure S24. ^{13}C-NMR spectrum of compound **4j**.

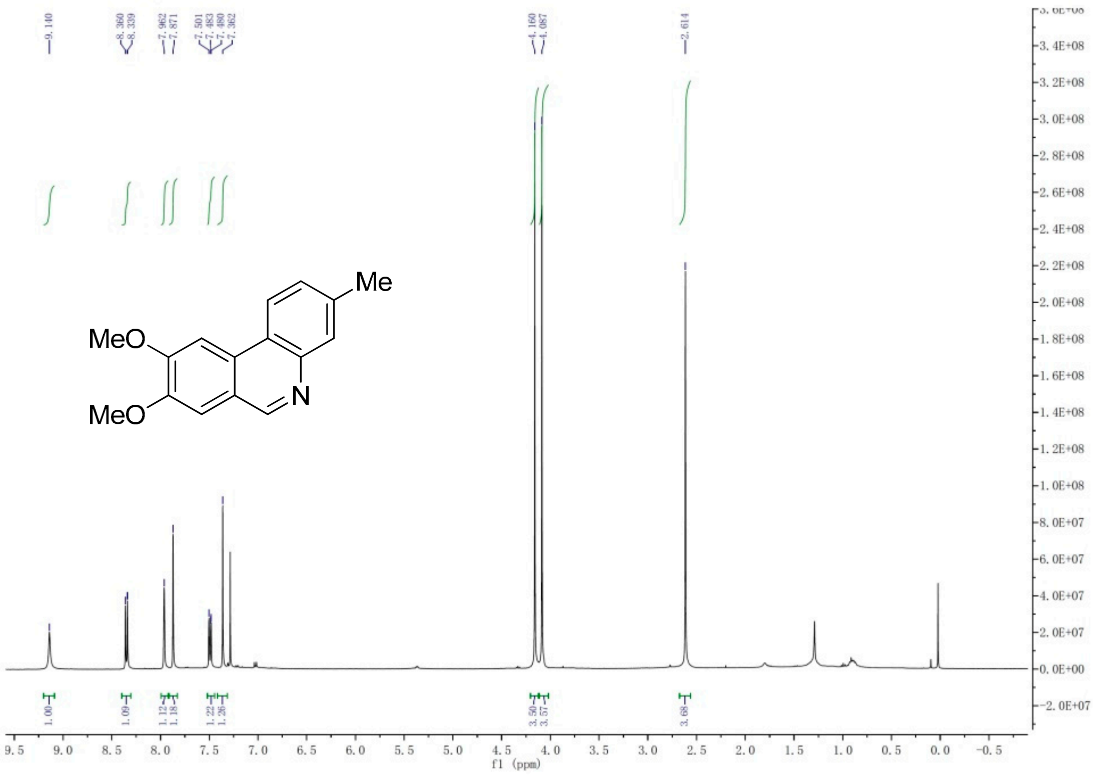

Figure S25. ^{1}H-NMR spectrum of compound **4k**.

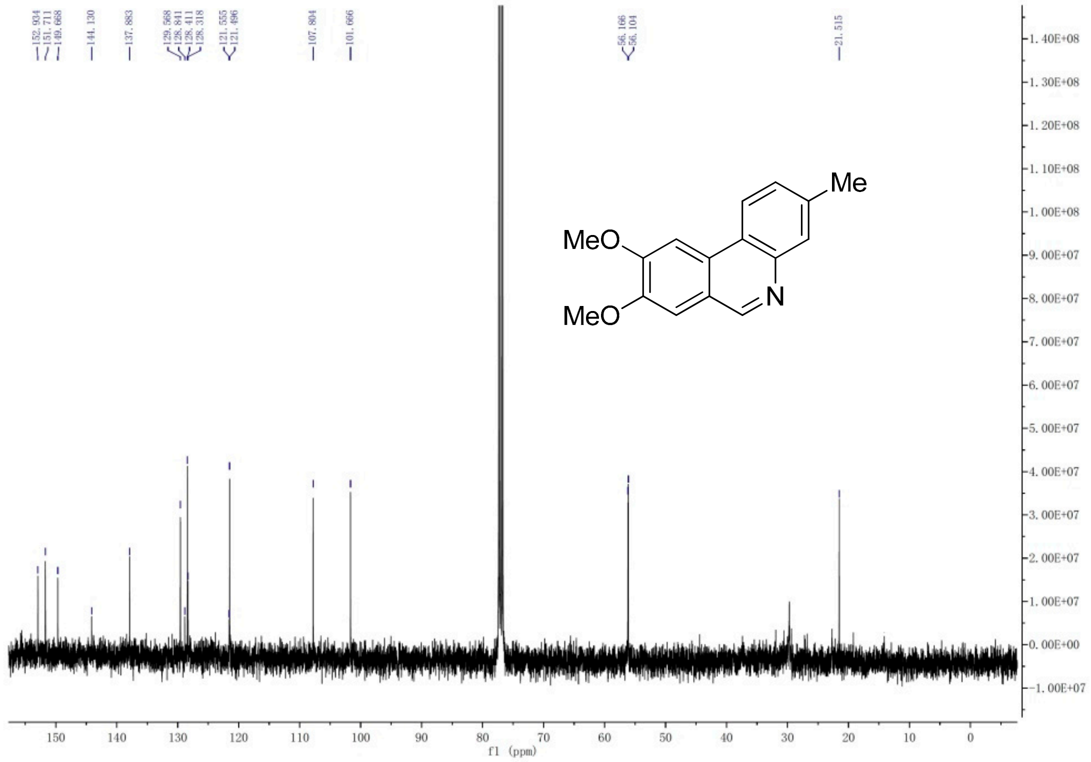

Figure S26. ^{13}C-NMR spectrum of compound **4k**.

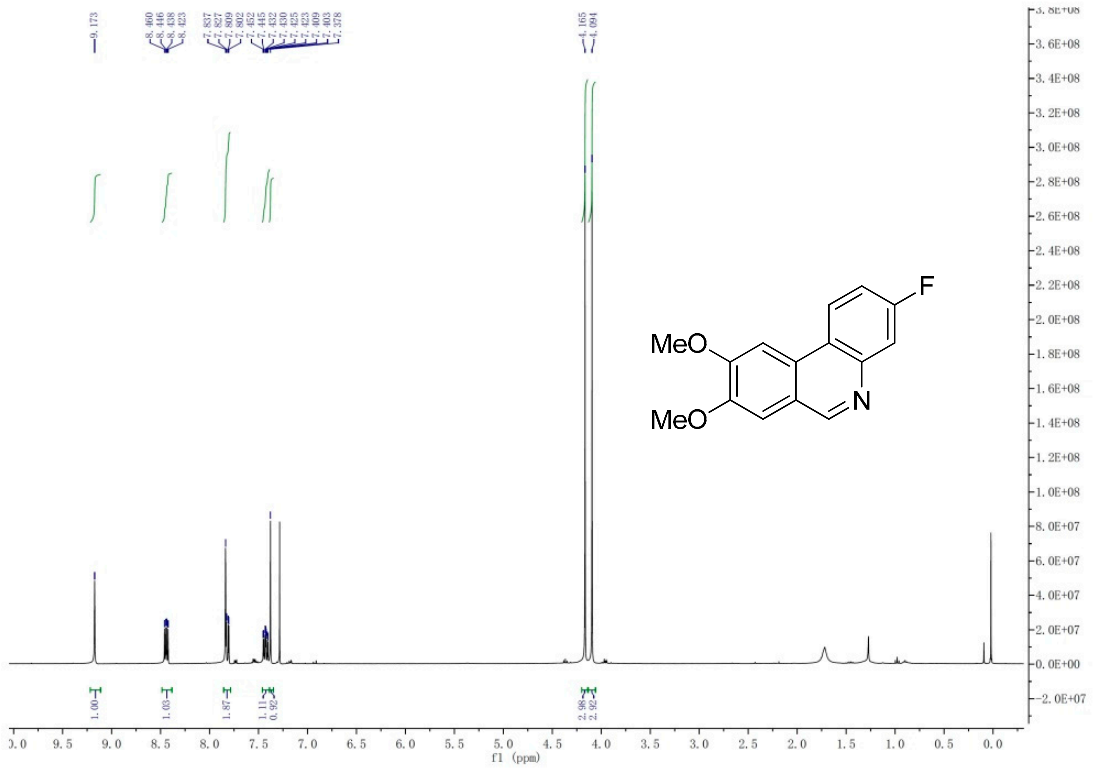

Figure S27. ^{1}H-NMR spectrum of compound **4l**.

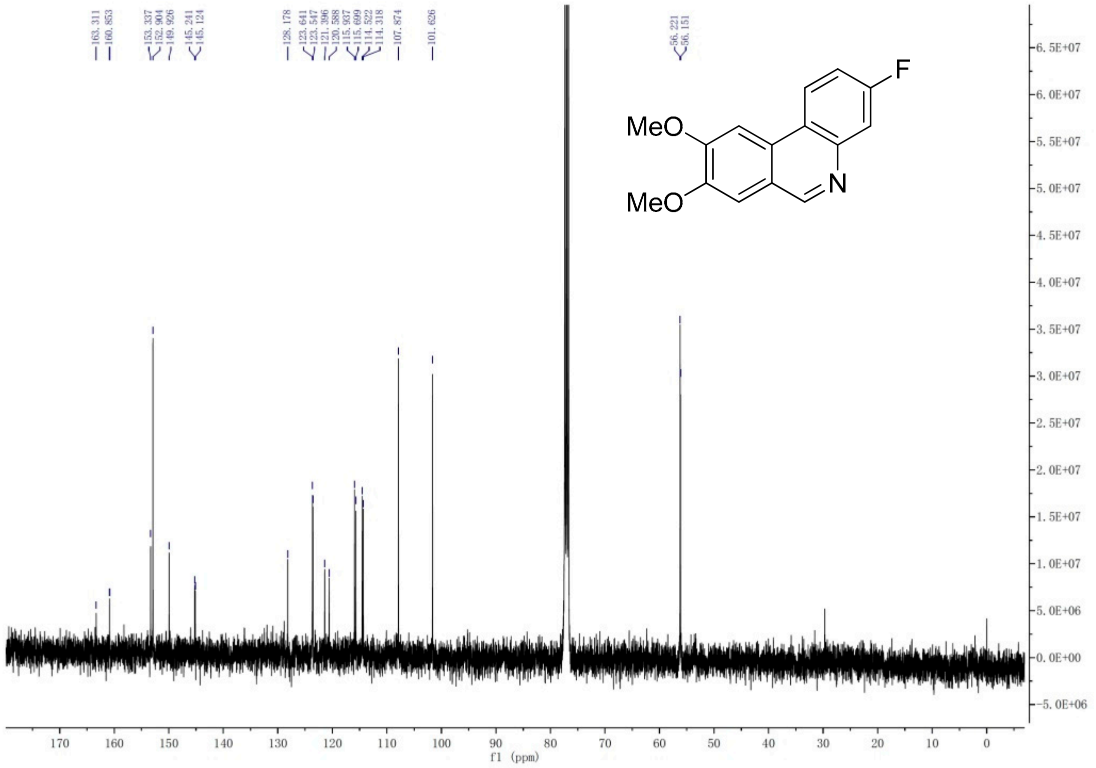

Figure S28. ^{13}C-NMR spectrum of compound **4l**.

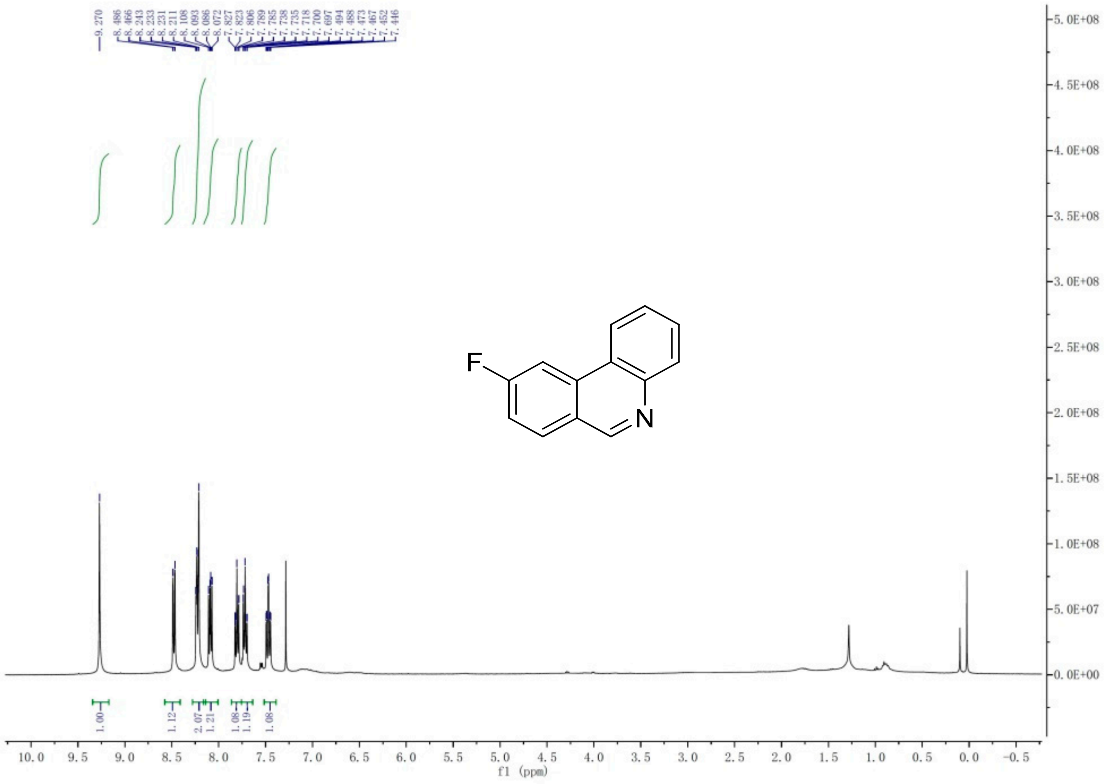

Figure S29. ^{1}H-NMR spectrum of compound **4m**.

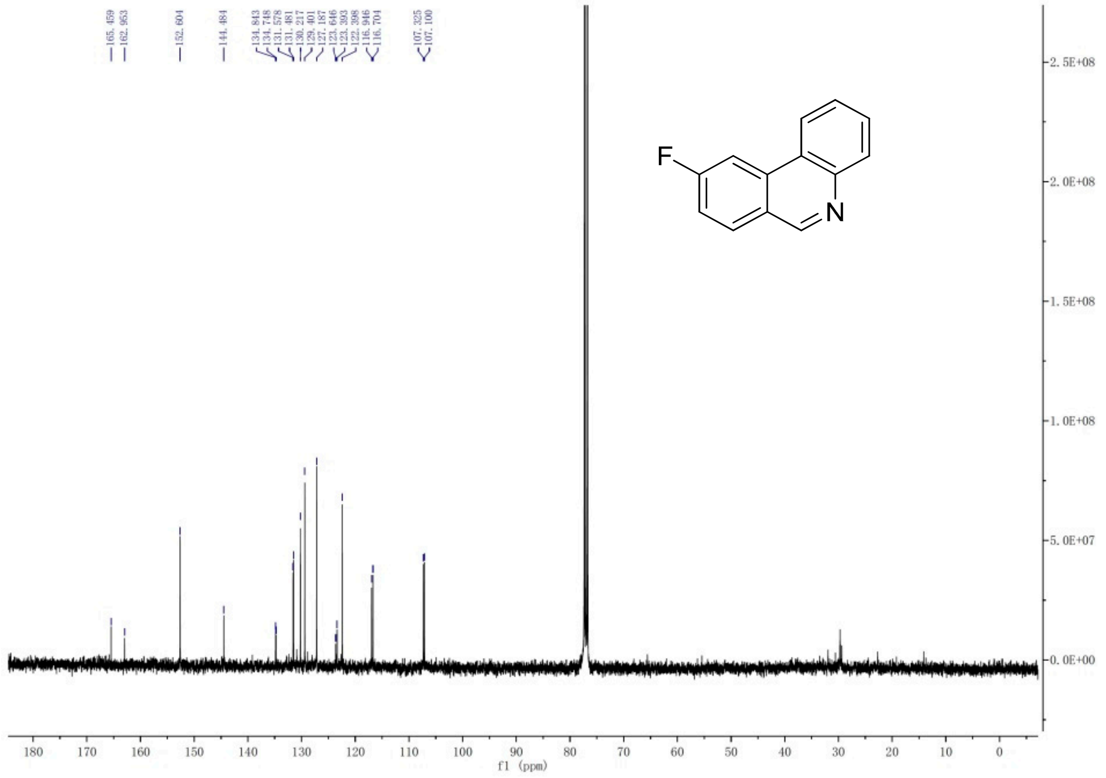

Figure S30. ^{13}C-NMR spectrum of compound **4m.**

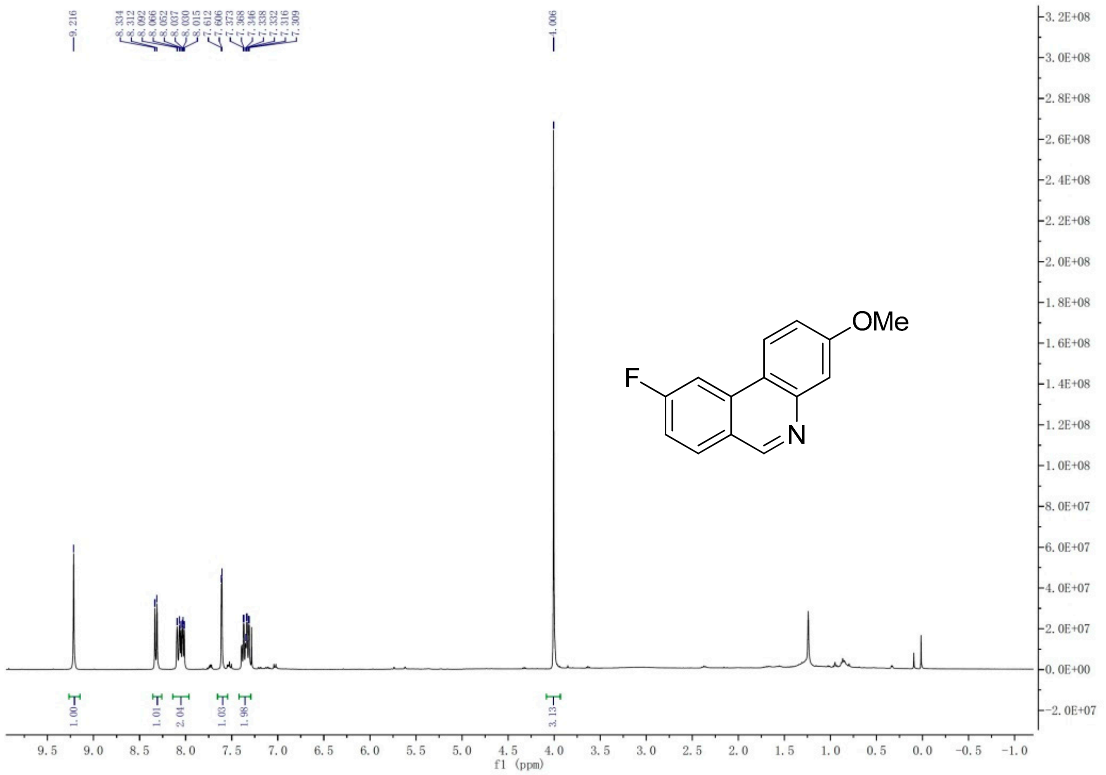

Figure S31. ^{1}H-NMR spectrum of compound **4n**.

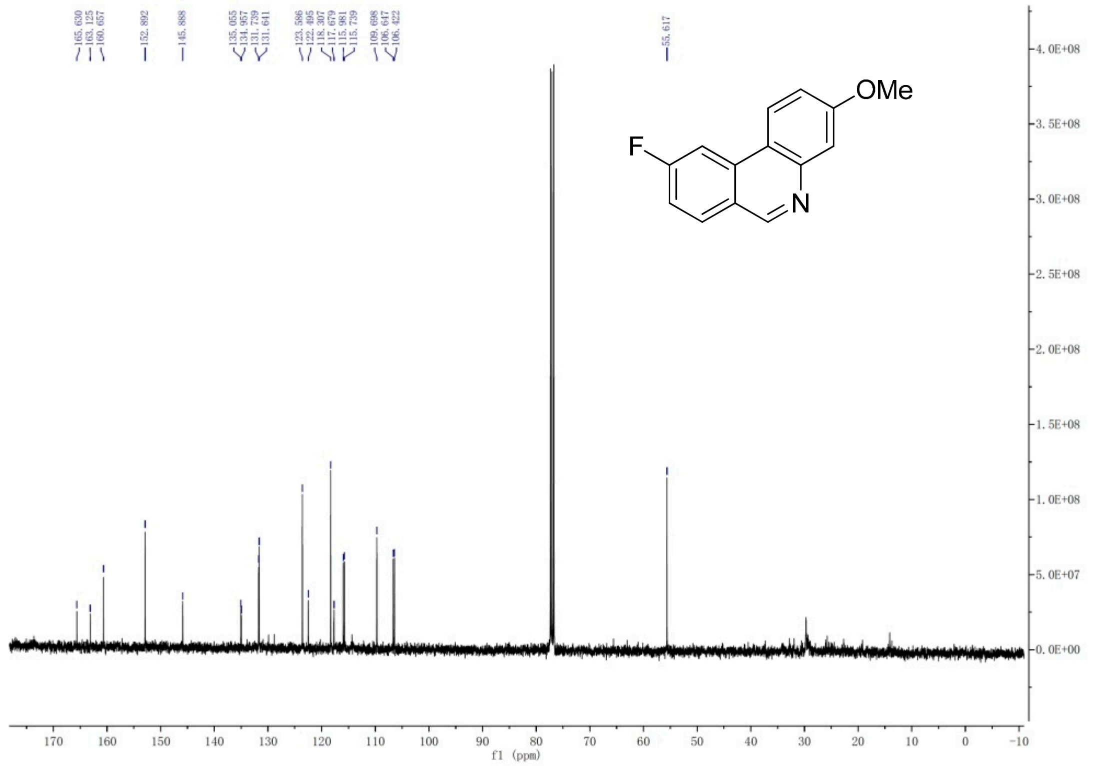

Figure S32. ^{13}C-NMR spectrum of compound **4n.**

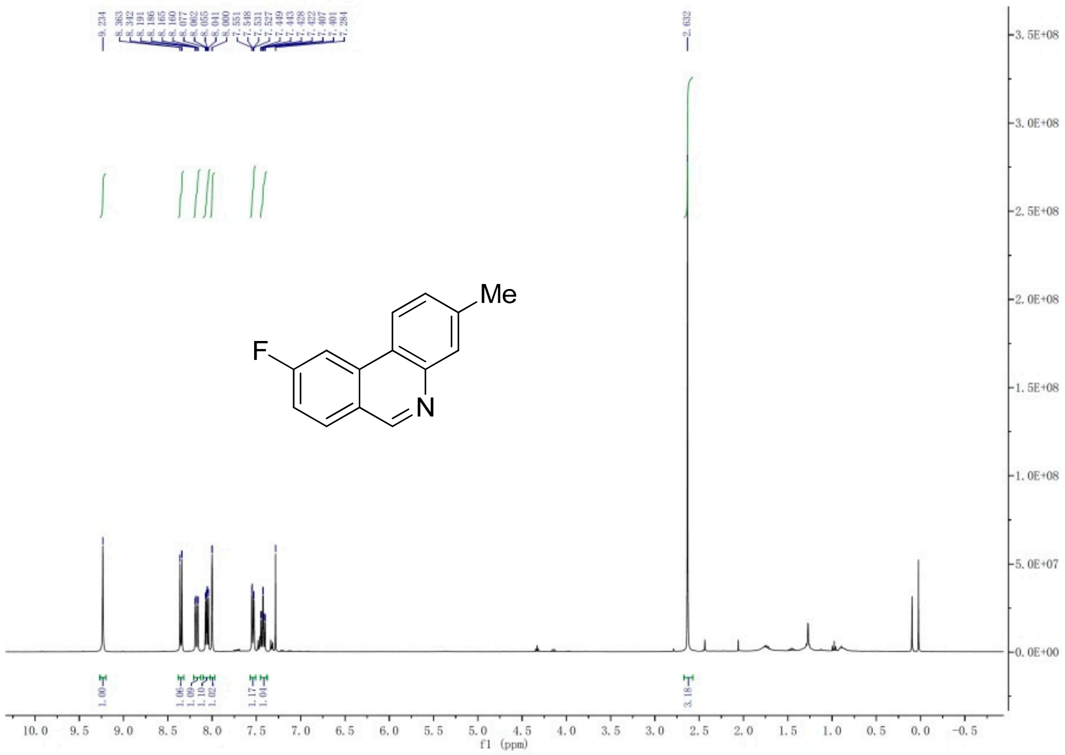

Figure S33. ^{1}H-NMR spectrum of compound **4o**.

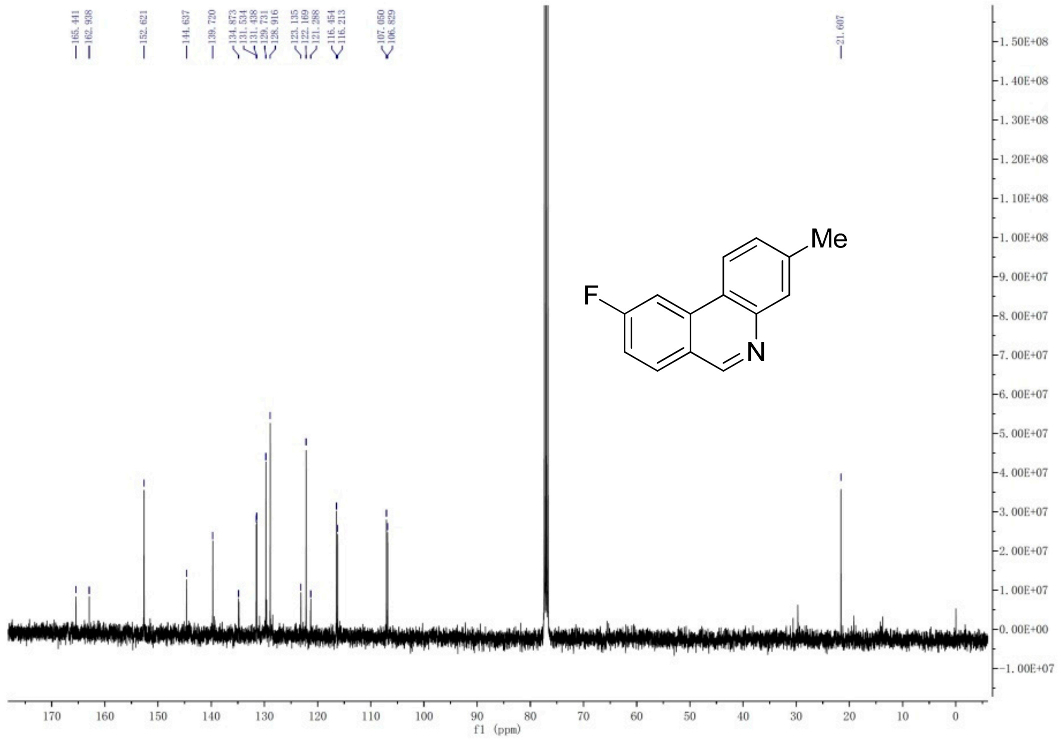

Figure S34. ^{13}C-NMR spectrum of compound **4o.**

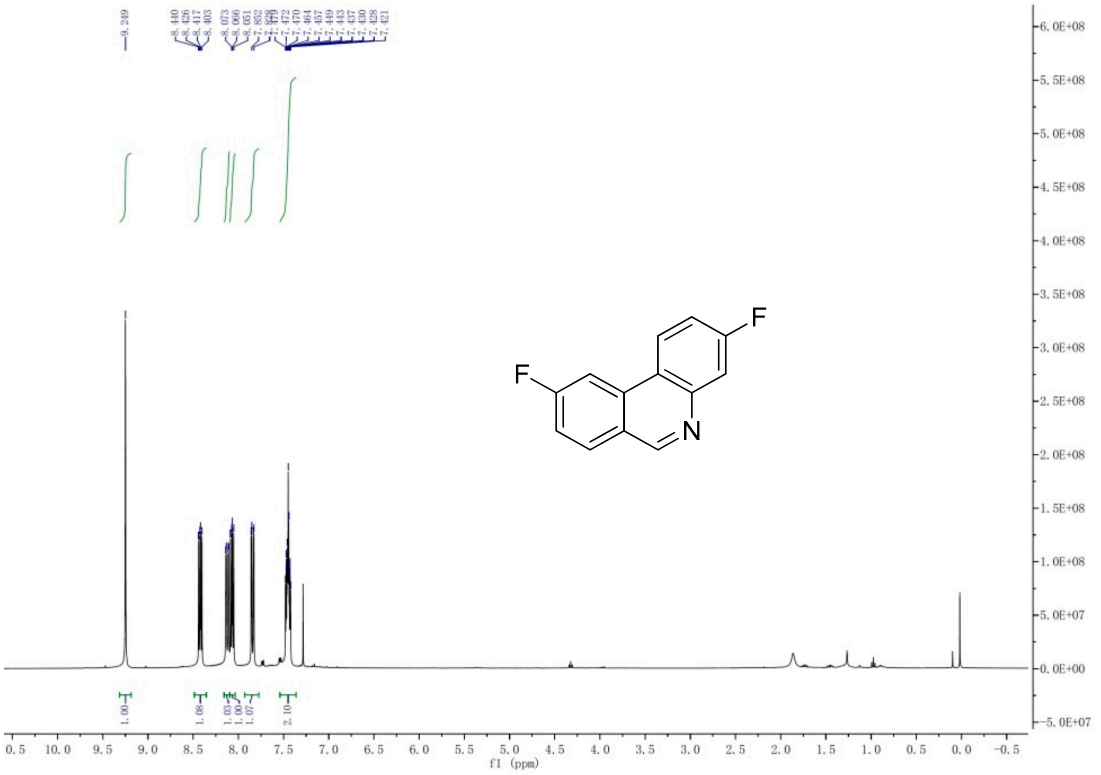

Figure S35. ^{1}H-NMR spectrum of compound **4p**.

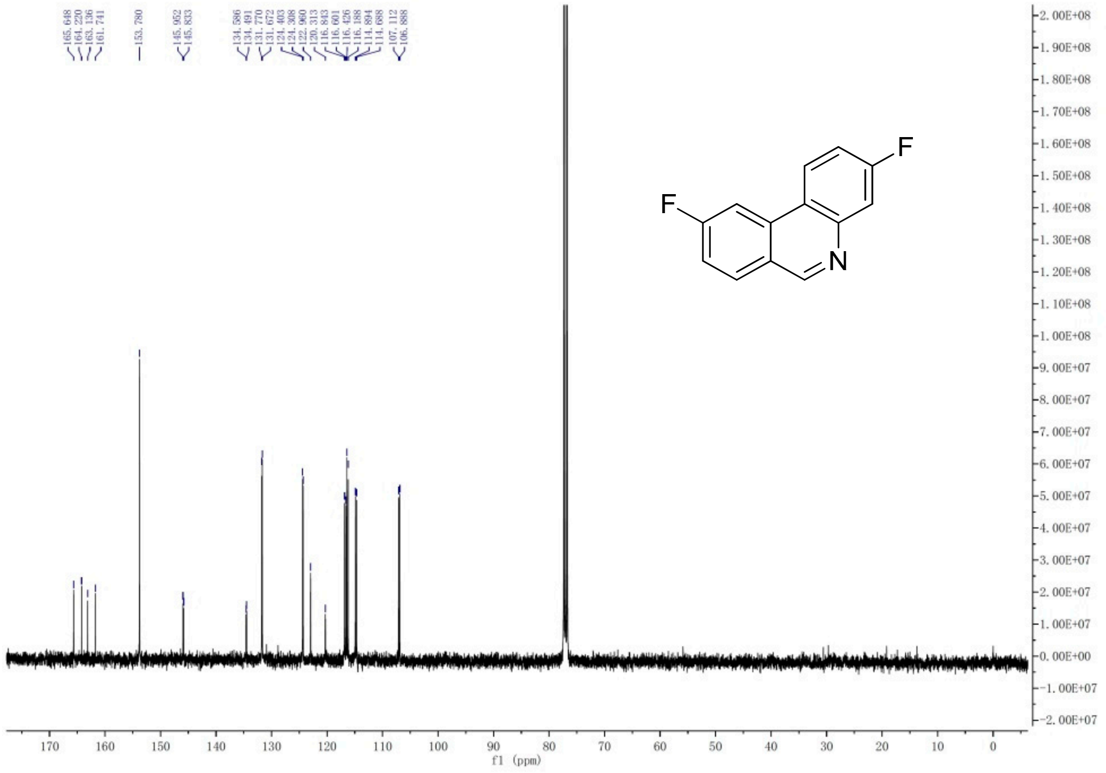

Figure S36. ^{13}C-NMR spectrum of compound **4p.**

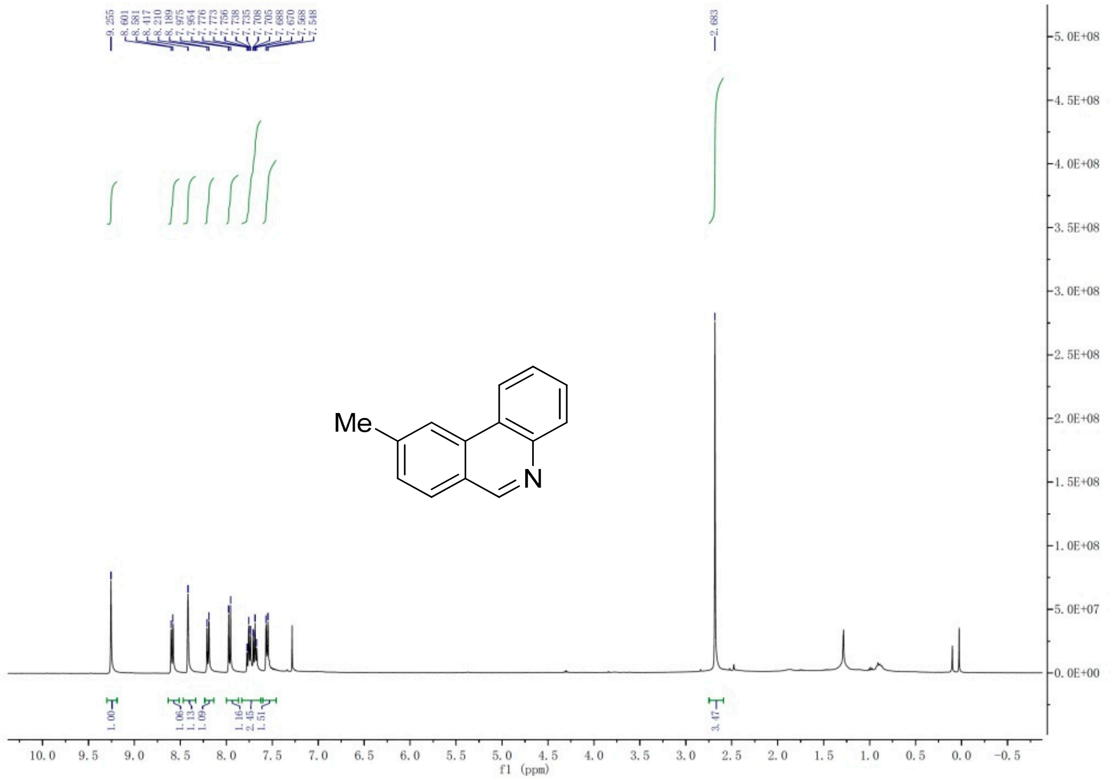

Figure S37. ^{1}H-NMR spectrum of compound **4q**.

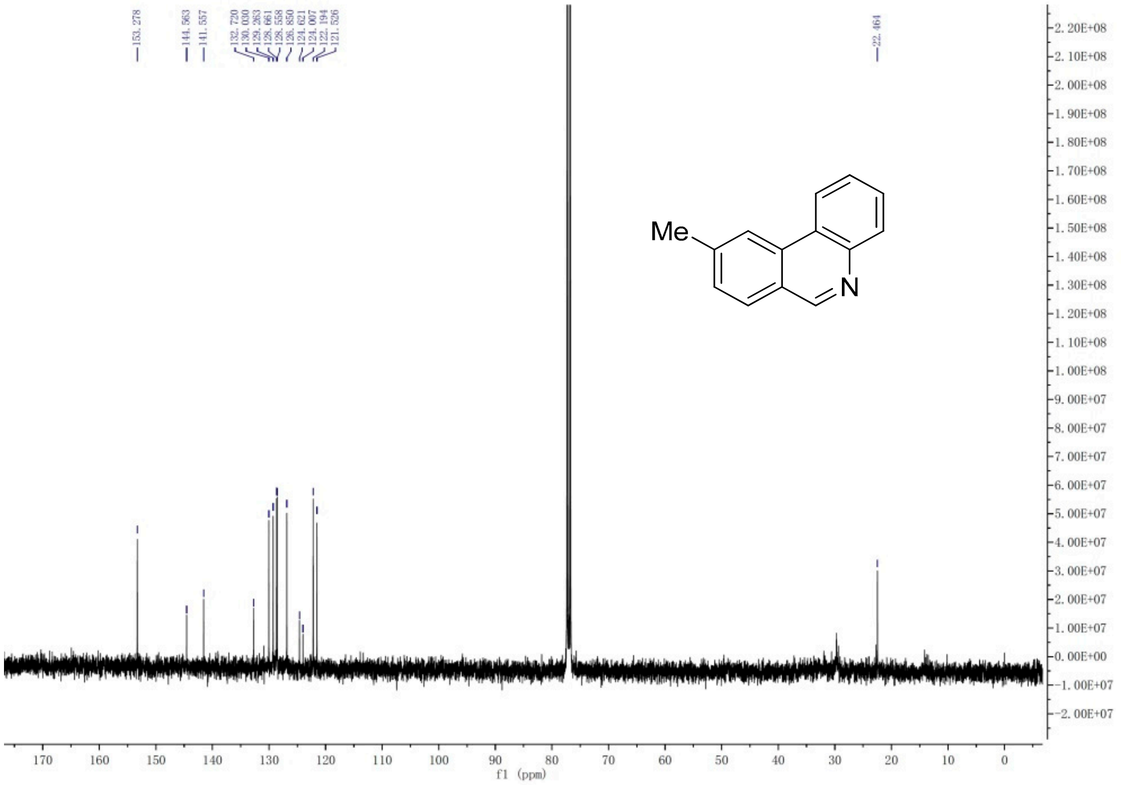

Figure S38. ^{13}C-NMR spectrum of compound **4q.**

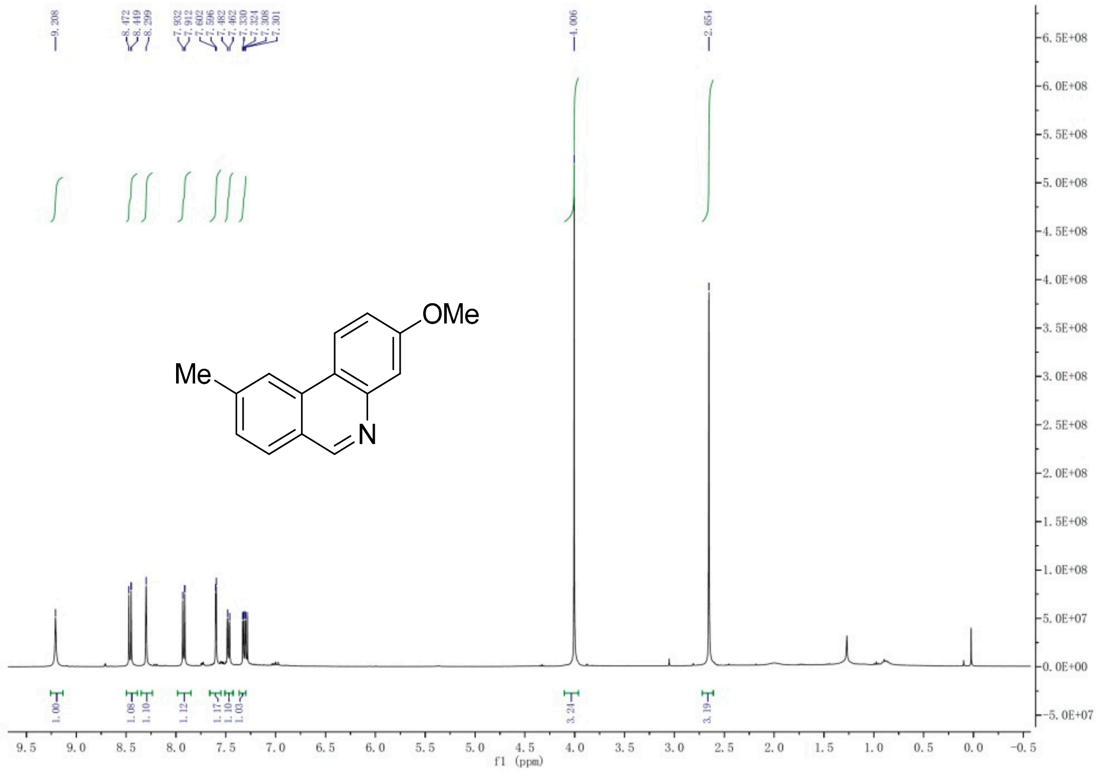

Figure S39. ^{1}H-NMR spectrum of compound **4r**.

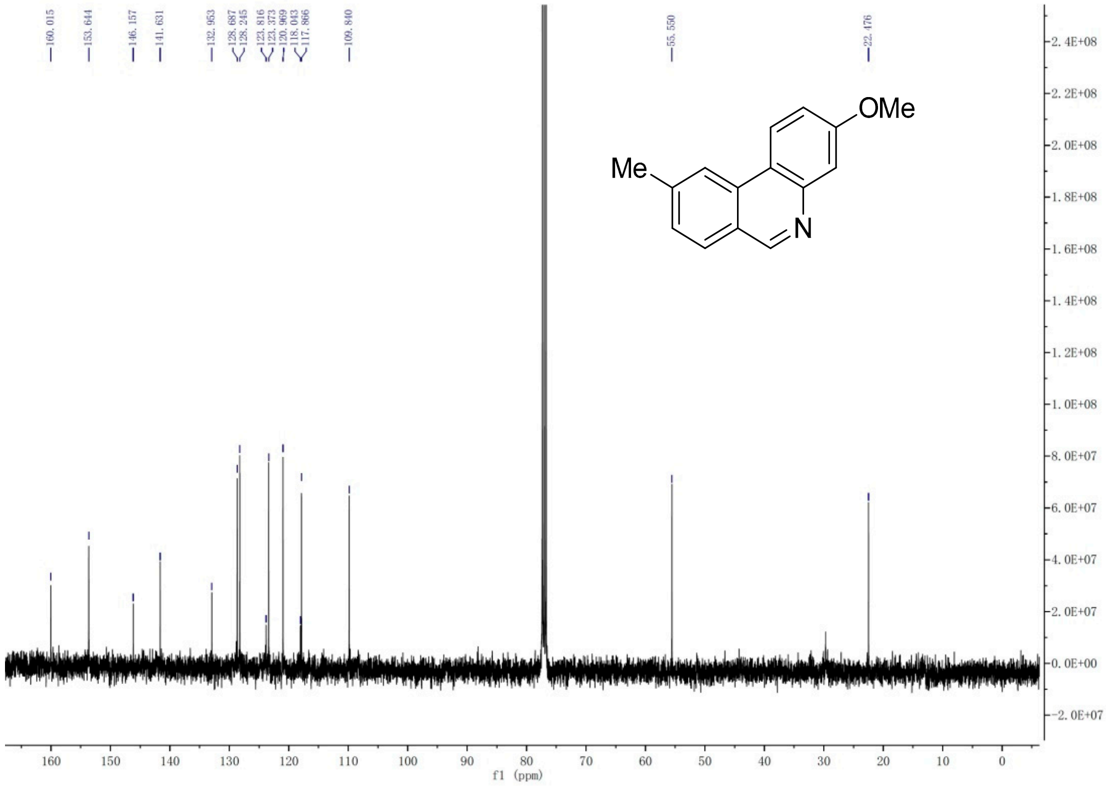

Figure S40. ^{13}C-NMR spectrum of compound **4r.**

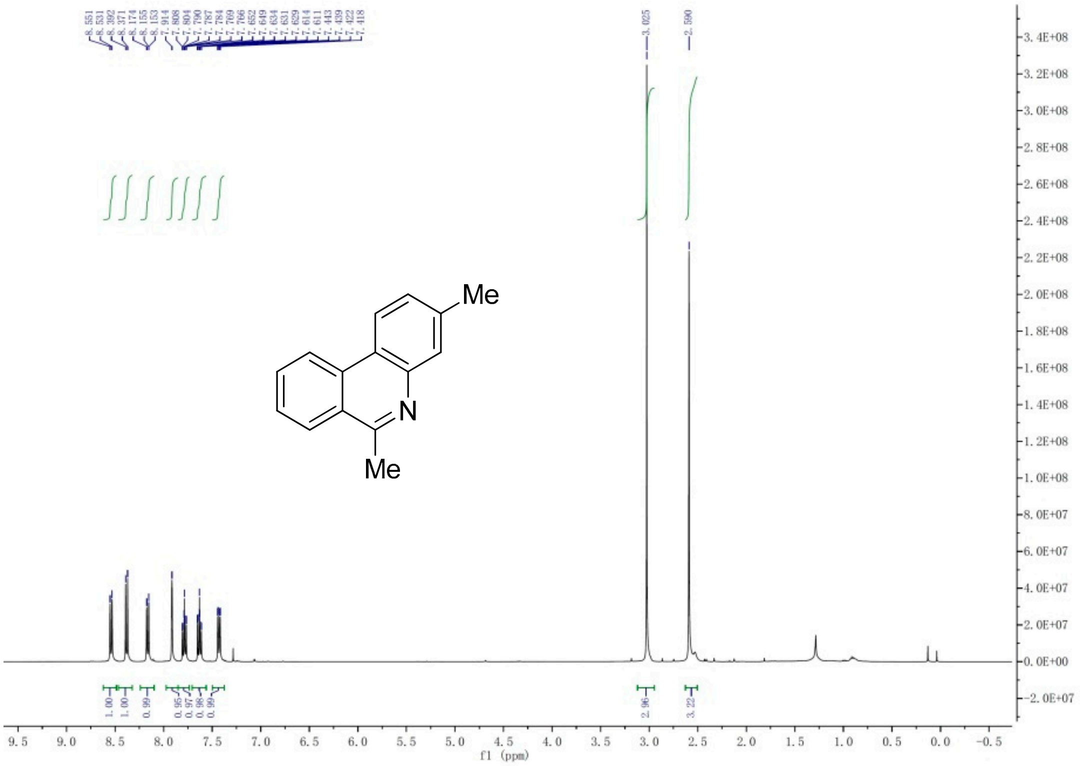

Figure S41. ^{1}H-NMR spectrum of compound **7b**.

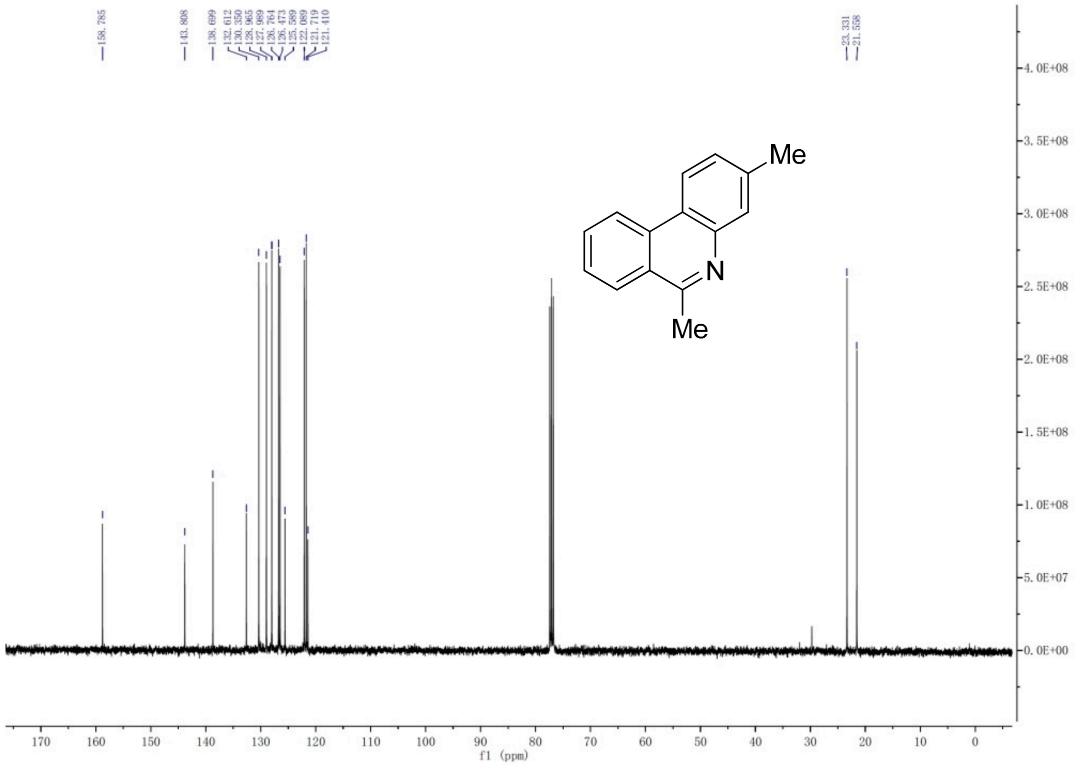

Figure S42. ^{13}C-NMR spectrum of compound **7b.**

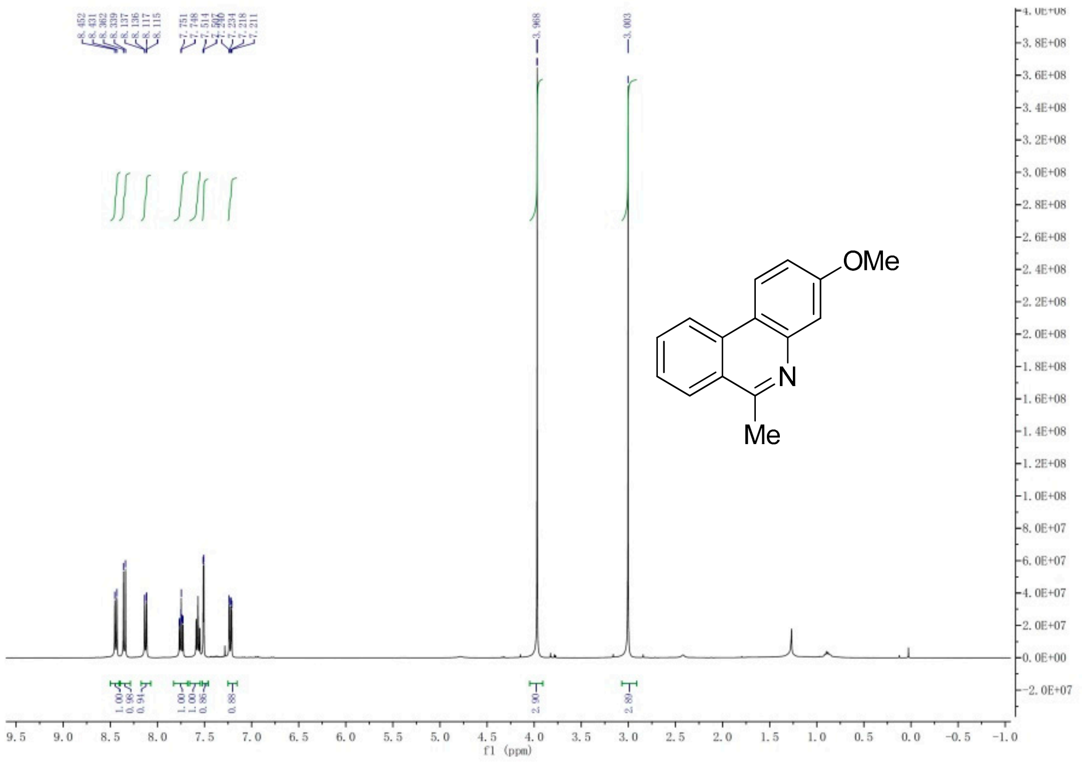

Figure S43. ^{1}H-NMR spectrum of compound **7c**.

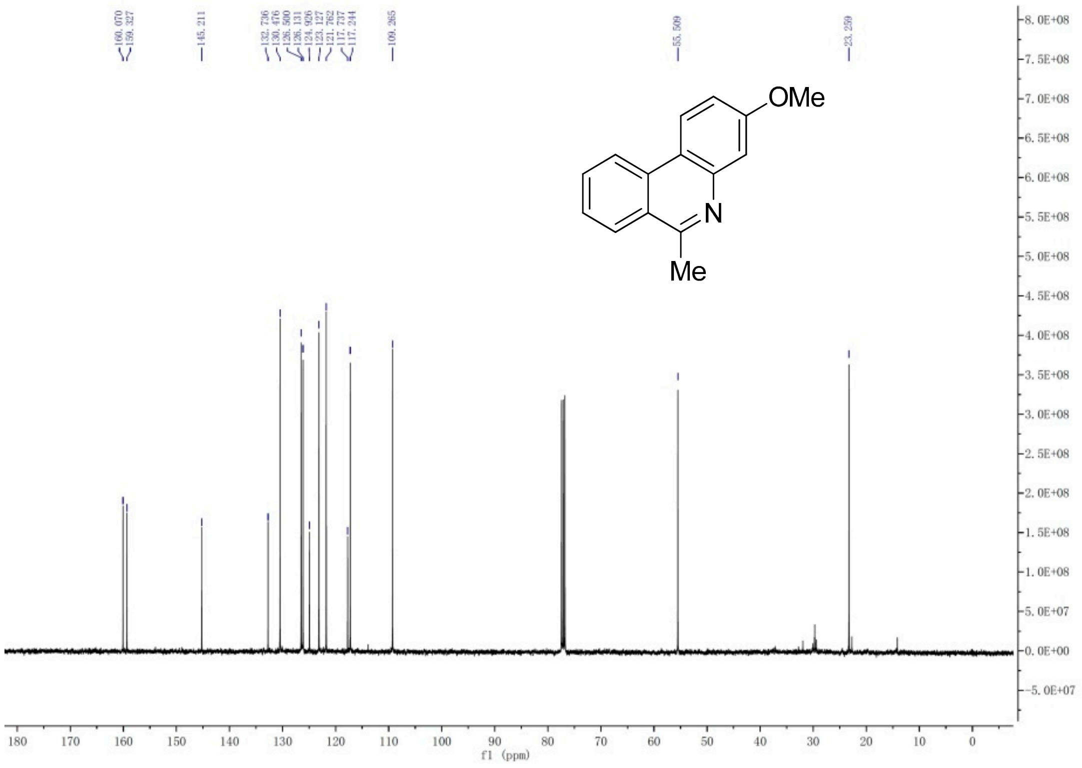

Figure S44. ^{13}C-NMR spectrum of compound **7c.**

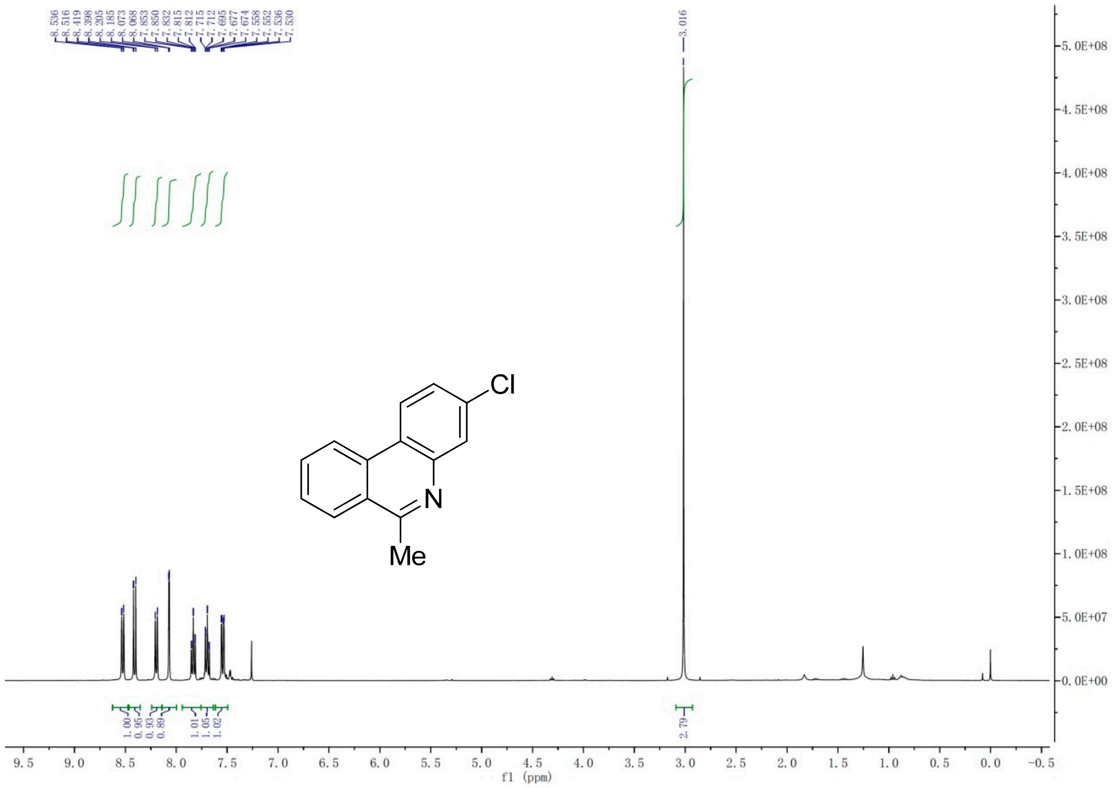

Figure S45. ¹H-NMR spectrum of compound **7d**.

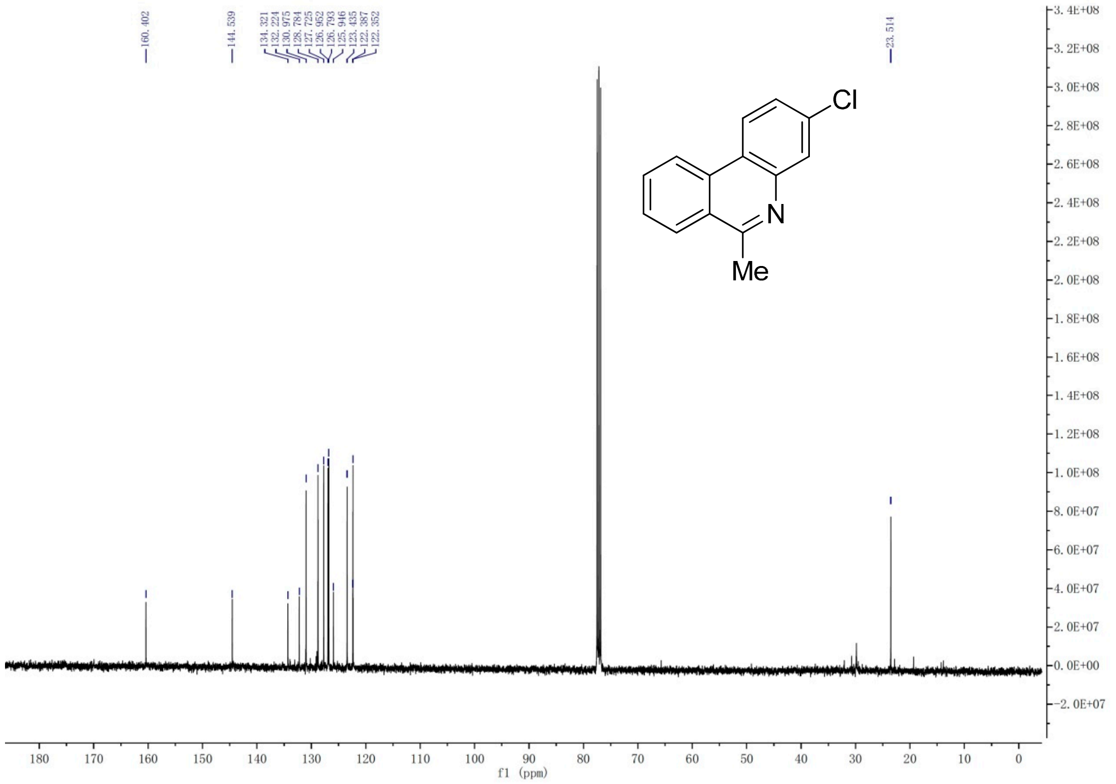

Figure S46. ¹³C-NMR spectrum of compound **7d.**

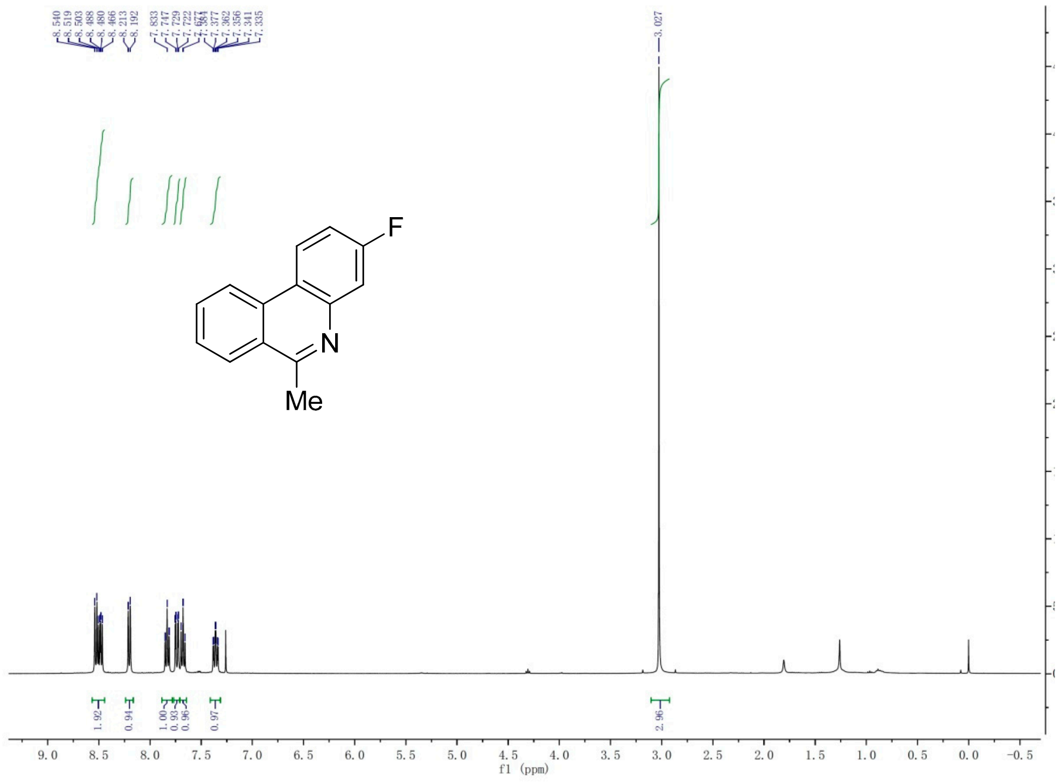

Figure S47. ^{1}H-NMR spectrum of compound **7e**.

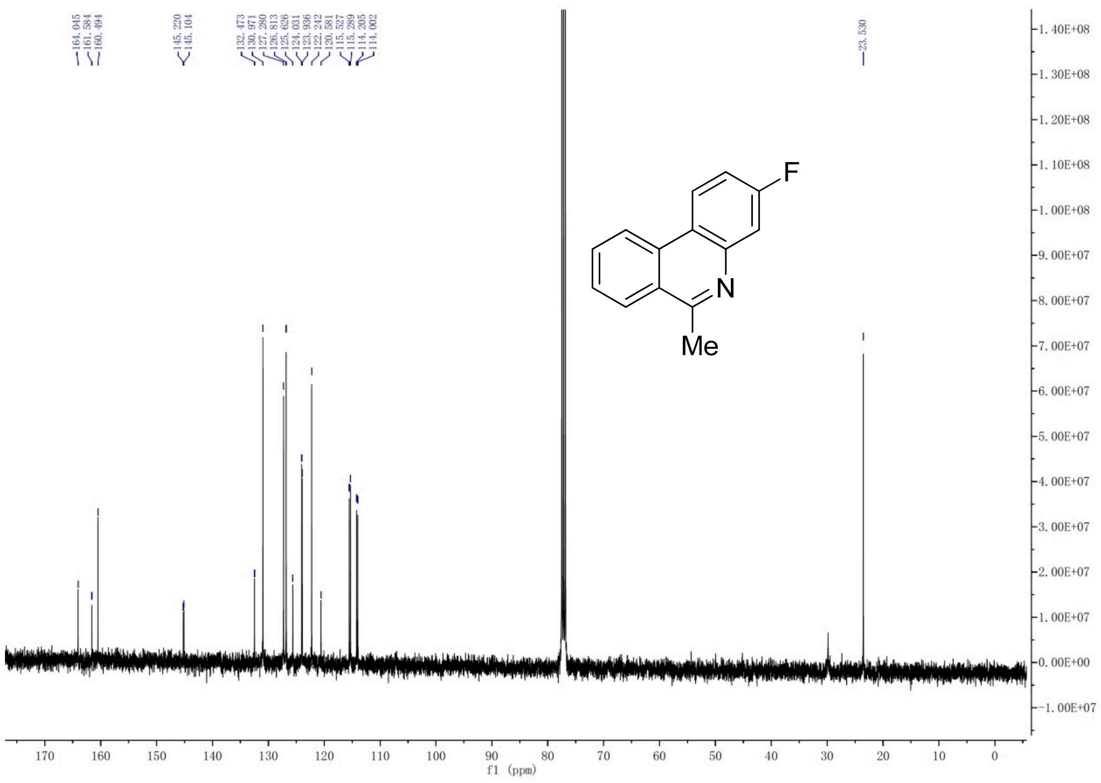

Figure S48. ^{13}C-NMR spectrum of compound **7e.**

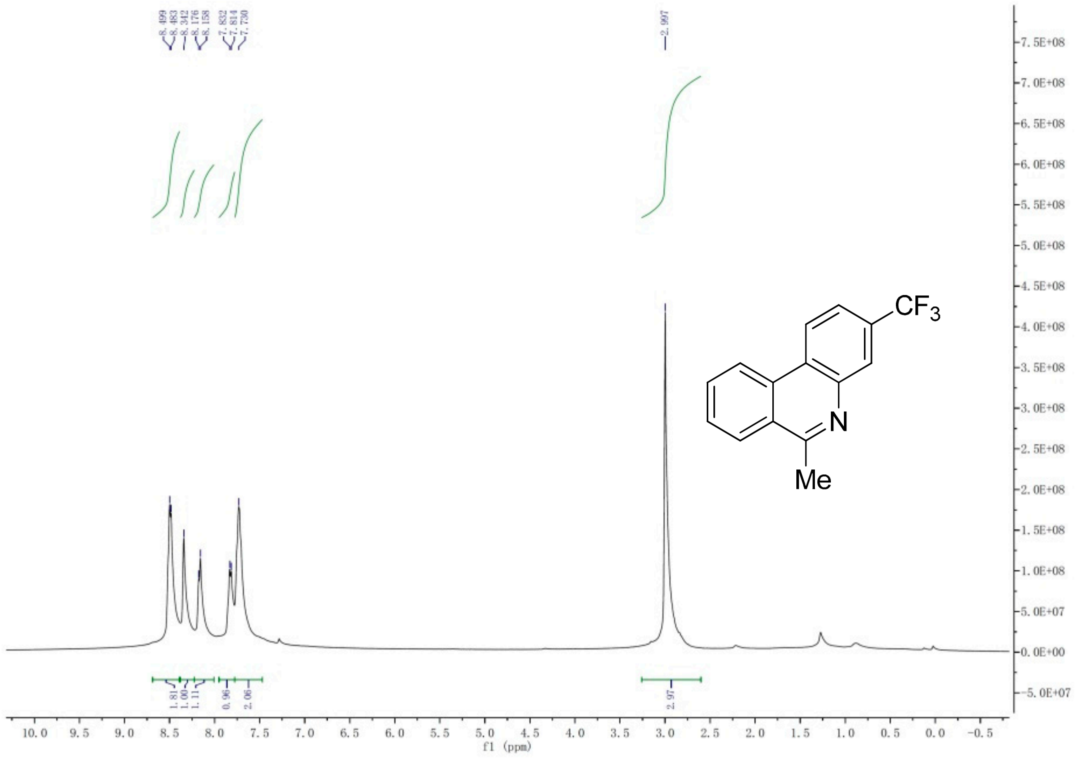

Figure S49. ^{1}H-NMR spectrum of compound **7f**.

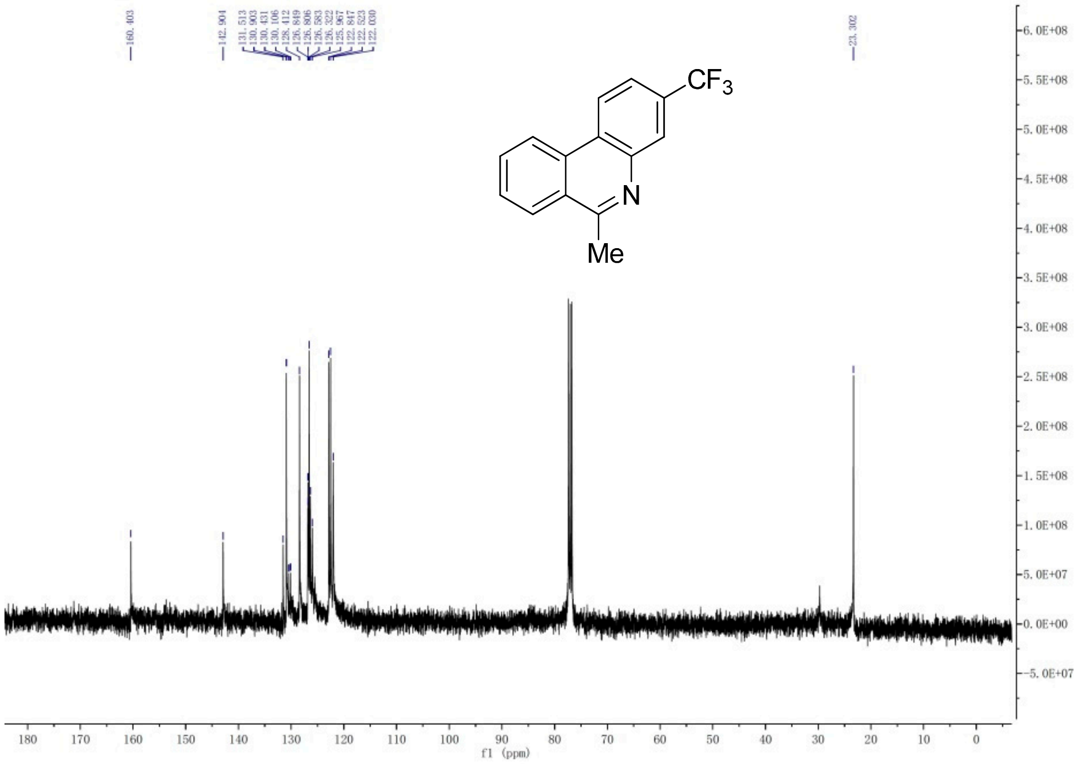

Figure S50. ^{13}C-NMR spectrum of compound **7f**
